# Regional desynchronization of microglial activity is associated with cognitive decline in Alzheimer’s disease

**DOI:** 10.1186/s13024-024-00752-6

**Published:** 2024-09-05

**Authors:** Artem Zatcepin, Johannes Gnörich, Boris-Stephan Rauchmann, Laura M. Bartos, Stephan Wagner, Nicolai Franzmeier, Maura Malpetti, Xianyuan Xiang, Yuan Shi, Samira Parhizkar, Maximilian Grosch, Karin Wind-Mark, Sebastian T. Kunte, Leonie Beyer, Carolin Meyer, Desirée Brösamle, Ann-Christin Wendeln, Collins Osei-Sarpong, Steffanie Heindl, Arthur Liesz, Sophia Stoecklein, Gloria Biechele, Anika Finze, Florian Eckenweber, Simon Lindner, Axel Rominger, Peter Bartenstein, Michael Willem, Sabina Tahirovic, Jochen Herms, Katharina Buerger, Mikael Simons, Christian Haass, Rainer Rupprecht, Markus J. Riemenschneider, Nathalie L. Albert, Marc Beyer, Jonas J. Neher, Lars Paeger, Johannes Levin, Günter U. Höglinger, Robert Perneczky, Sibylle I. Ziegler, Matthias Brendel

**Affiliations:** 1 Department of Nuclear Medicine, University Hospital, LMU Munich, Munich, Germany; 2 German Center for Neurodegenerative Diseases (DZNE), Munich, Germany; 3 Institute of Neuroradiology, University Hospital LMU, Munich, Germany; 4 Department of Psychiatry and Psychotherapy, University Hospital, LMU Munich, Munich, Germany; 5 Institute for Stroke and Dementia Research, University Hospital of Munich, LMU Munich, Munich, Germany; 6 Department of Psychiatry and Neurochemistry, Institute of Neuroscience and Physiology, The Sahlgrenska Academy, University of Gothenburg, Gothenburg, Sweden; 7 Munich Cluster for Systems Neurology (SyNergy), Munich, Germany; 8 Department of Clinical Neurosciences, Cambridge University Hospitals NHS Trust, University of Cambridge, Cambridge, UK; 9 Biomedical Center (BMC), Division of Metabolic Biochemistry, Faculty of Medicine, LMU Munich, Munich, Germany; 10 CAS Key Laboratory of Brain Connectome and Manipulation, the Brain Cognition and Brain Disease Institute, Institutes of Advanced Technology, Chinese Academy of Sciences, Shenzhen-Hong Kong Institute of Brain Science-Shenzhen Fundamental Research Institutions, ShenzhenShenzhen, 518055 China; 11 Department of Neurology, Washington University in St. Louis, St. Louis, MO USA; 12 German Center for Vertigo and Balance Disorders, University Hospital of Munich, LMU Munich, Munich, Germany; 13 German Center for Neurodegenerative Disease (DZNE), Neuroimmunology and Neurodegenerative Diseases, Göttingen, Germany; 14 Dept. of Cellular Neurology, Hertie Institute for Clinical Brain Research, Tübingen, Germany; 15 Metabolic Biochemistry, Faculty of Medicine, Biomedical Center Munich (BMC), LMU Munich, Munich, Germany; 16 Platform for Single Cell Genomics and Epigenomics (PRECISE), German Center for Neurodegenerative Diseasesand , University of Bonn and West German Genome Center, Bonn, Germany; 17 German Center for Neurodegenerative Diseases (DZNE), Immunogenomics & Neurodegeneration, Bonn, Germany; 18 Department of Radiology, University Hospital, LMU Munich, Munich, Germany; 19 Department of Nuclear Medicine, Inselpital, Bern University Hospital, University of Bern, Bern, Switzerland; 20 Center for Neuropathology and Prion Research, LMU Munich, Munich, Germany; 21 Institute of Neuronal Cell Biology, Technical University Munich, Munich, Germany; 22 Department of Psychiatry and Psychotherapy, University of Regensburg, Molecular Neurosciences, Regensburg, Germany; 23 Department of Neuropathology, Regensburg University Hospital, Regensburg, Germany; 24 German Cancer Research Center (DKFZ), German Cancer Consortium (DKTK), Partner Site Munich, 69120 Heidelberg, Germany; 25 Bavarian Cancer Research Center (BZKF), 91054 Erlangen, Germany; 26 Department of Neurology, University Hospital, LMU Munich, Munich, Germany; 27 Department of Neurology, Hannover Medical School, Carl-Neuberg-Str. 1, 30625 Hannover, Germany; 28 Ageing Epidemiology (AGE) Research Unit, School of Public Health, Imperial College London, London, W6 8RP UK; 29 Sheffield Institute for Translational Neuroscience (SITraN), University of Sheffield, Sheffield, S10 2HQ UK

**Keywords:** Alzheimer’s disease, Dementia, Microglia, Neuroinflammation, TSPO, PET, Brain connectivity, Microglia synchronicity, Microglia desynchronization

## Abstract

**Background:**

Microglial activation is one hallmark of Alzheimer disease (AD) neuropathology but the impact of the regional interplay of microglia cells in the brain is poorly understood. We hypothesized that microglial activation is regionally synchronized in the healthy brain but experiences regional desynchronization with ongoing neurodegenerative disease. We addressed the existence of a microglia connectome and investigated microglial desynchronization as an AD biomarker.

**Methods:**

To validate the concept, we performed microglia depletion in mice to test whether interregional correlation coefficients (ICCs) of 18 kDa translocator protein (TSPO)-PET change when microglia are cleared. Next, we evaluated the influence of dysfunctional microglia and AD pathophysiology on TSPO-PET ICCs in the mouse brain, followed by translation to a human AD-continuum dataset. We correlated a personalized microglia desynchronization index with cognitive performance. Finally, we performed single-cell radiotracing (scRadiotracing) in mice to ensure the microglial source of the measured desynchronization.

**Results:**

Microglia-depleted mice showed a strong ICC reduction in all brain compartments, indicating microglia-specific desynchronization. AD mouse models demonstrated significant reductions of microglial synchronicity, associated with increasing variability of cellular radiotracer uptake in pathologically altered brain regions. Humans within the AD-continuum indicated a stage-depended reduction of microglia synchronicity associated with cognitive decline. scRadiotracing in mice showed that the increased TSPO signal was attributed to microglia.

**Conclusion:**

Using TSPO-PET imaging of mice with depleted microglia and scRadiotracing in an amyloid model, we provide first evidence that a microglia connectome can be assessed in the mouse brain. Microglia synchronicity is closely associated with cognitive decline in AD and could serve as an independent personalized biomarker for disease progression.

**Supplementary Information:**

The online version contains supplementary material available at 10.1186/s13024-024-00752-6.

## Background

Microglial activation constitutes a hallmark in the pathophysiology of Alzheimer’s disease (AD) [1] and has been associated with aggregation of misfolded β-amyloid (Aβ) and tau accumulation in patients with AD and in mouse models of AD [2]. Moreover, the levels of microglial activation follow stage-dependent trajectories [3, 4] and have close associations with fibrillar tau aggregates [5, 6]. Triggering receptor expressed on myeloid cells 2 (TREM2) in cerebrospinal fluid (CSF) is a promising biomarker of microglial activation in vivo, which indicated protective effects on brain atrophy and cognitive performance during early stages of AD [7, 8] and is related to slower amyloid accumulation [9]. However, microglial activation is characterized by a variety of disease-associated (DAM) phenotypes, including TREM2-dependent and independent pathways in AD [10] and the spectrum of activated microglia is also subject to regional heterogeneity [11]. In this regard, assessment of microglial activation with positron emission tomography (PET) provides the opportunity to measure region-dependent alterations by means of 18 kDa translocator protein (*TSPO*) expression [12]. Multiple preclinical and clinical studies found region-specific TSPO-PET increases in patients with AD [13] and models of AD neuropathology [14]; moreover, increased TSPO could be detected in microglial proteome of AD mouse models (APPPS1 and APP-KI) [15]. However, several aspects are still unsolved, such as inconsistent results in proposed target regions, regional heterogeneity of the TSPO-PET signal, and suggested multiphasic trajectories during the disease course [3, 16], which results in the lack of a standardized approach to analyze TSPO-PET. Thus, lacking a harmonized and personalized TSPO-PET index strongly hampered the use in clinical trials of immunomodulatory therapies in AD.

Given the large heterogeneity of DAM states, regional differences of microglia phenotypes and topological heterogeneity of AD [17], we hypothesized that microglial activation is regionally synchronized in the healthy brain but experiences regional desynchronization with ongoing neurodegenerative disease. To obtain an index of microglia synchronicity, we transferred the strategy of quantifying and thresholding (i.e., number of significant connections) interregional correlations from research on metabolic connectivity [18]. Thus, we defined microglia synchronicity in this work on a cohort level as the aggregate of the cohort’s interregional correlation coefficients (ICCs), while the reduction of the cohort’s ICCs is referred to as microglia desynchronization. On a subject level, microglia desynchronization is defined in this work as a magnitude of deviation from the microglia synchronicity of a normal cohort. We used microglia depletion in mice that were then scanned with in vivo [^18^F]GE-180 PET, a tracer that binds to TSPO, to investigate if microglia synchronicity can be measured via TSPO-PET and if this read-out yields potential as an AD biomarker. Furthermore, we investigated microglia synchronicity in different mouse models of AD (Aβ and tau). Moreover, we translated our approach into a patient cohort with AD and evaluated whether microglial desynchronization is associated with their disease stage and cognitive decline. Finally, we used cell sorting after in vivo radiotracer injection to determine the biological source of regionally desynchronized TSPO tracer uptake. Thus, this approach provided a unique insight into the biology and pathophysiology of microglia in preclinical AD models and AD patients.

## Methods

### Experimental design

**Fig. 1:**
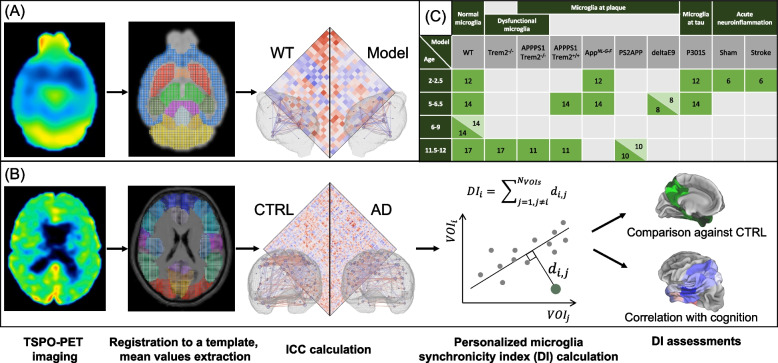
Study design. In both mouse (**A**) and human (**B**) studies, TSPO-PET images were registered to a tracer specific template. Based on extracted mean values, inter-correlation-coefficients (ICCs) were calculated. In the human study (**B**), we additionally calculated a microglia synchronicity index (desynchronization index, DI) for each subject on a single volume-of-interest (VOI) basis (see detailed explanation further in text and in Fig. 9A). For each VOI, we compared the DIs between studied cohorts and correlated it with two cognition scores. **C** Overview of mouse cohorts. The numbers in green indicate the number of mice in corresponding cohorts. Light green color stands for mice with pharmacologically depleted microglia (PLX5622). WT = wild type; AD = Alzheimer’s disease; CTRL = healthy control

This translational study used preclinical (*n* = 224) and human (*n* = 59) TSPO-PET imaging data, which were consistently analyzed with ICCs for assessment of regional synchronicity of the tracer signal (Fig. 1A and B). Mice with microglia depletion via colony-stimulating factor 1 receptor (CSF1R) inhibition using PLX5622 were analyzed in contrast to age-matched placebo controls as a proof-of-concept experiment. Regional synchronicity of TSPO-PET data was investigated in mice with microglia dysfunction and in AD mouse models of Aβ or tau pathology to recapitulate key AD-related brain changes. Human TSPO-PET scans were obtained from the ActiGliA cohort study [4], and we analyzed ICC in dependence of AD stages and cognitive performance. We performed single-cell radiotracing (scRadiotracing) after radiotracer injection with forebrains and hindbrains of wild-type (WT) and AD mice to determine the cellular sources of TSPO-PET tracer binding and the biological basis of regional signal desynchronization.

### Animal experiments

The experiments have been approved by the local animal care committee of the Government of Upper Bavaria (Regierung Oberbayern, approval numbers: ROB-55.2–2532.Vet_02-15–210, ROB-55.2–2532.Vet_02-19–26), overseen by a veterinarian and in compliance with the ARRIVE guidelines and were carried out in accordance with the U.K. Animals (Scientific Procedures) Act, 1986 and associated guidelines, EU Directive 2010/63/EU for animal experiments. The ARRIVE essential ten checklist is provided with the manuscript. Animals were housed in a temperature- and humidity-controlled environment with 12 h light–dark cycle, with free access to food and water. Each mouse was treated as one individual. WT mice were used as controls. All the procedures were performed at the Department of Nuclear Medicine, LMU University Hospital, LMU Munich. An overview of all included small animal study groups is provided in Fig. 1C and Supplementary Table S1.

**Fig. 2:**
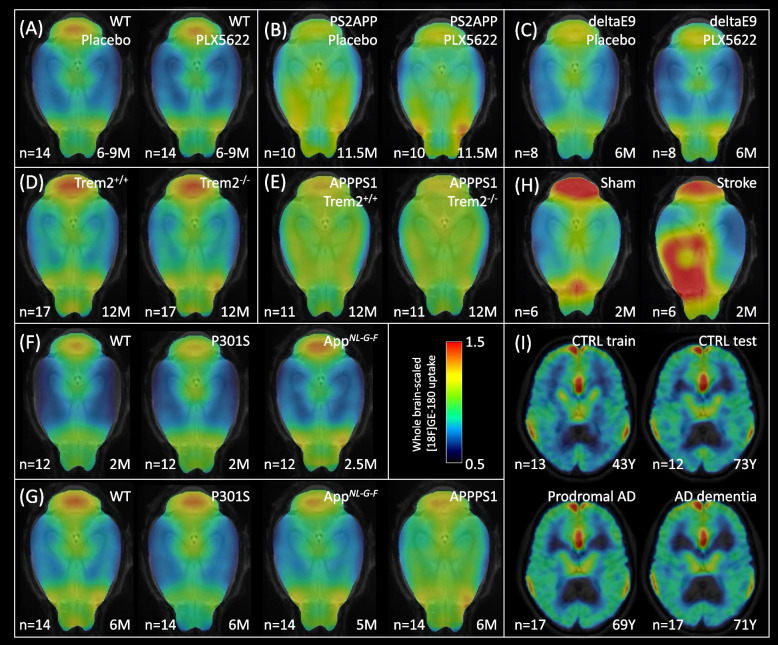
Mean TSPO-PET uptake (global mean-scaled) in each study cohort. Microglia depletion study in mice: **A** WT mice with microglia depleted by PLX5622 injection (WT PLX5622) and age-matched WT mice with placebo injection (WT Placebo) at 6–9 months of age, **B** Aβ mouse model (PS2APP) with microglia depleted by PLX5622 injection (PS2APP PLX5622) and age-matched mice with placebo injection (PS2APP Placebo) at 11.5 months of age, **C** Aβ mouse model (deltaE9) with microglia depleted by PLX5622 injection (deltaE9 PLX5622) and age-matched mice with placebo injection (deltaE9 Placebo) at 6 months of age.** D** Dysfunctional microglia study in mice: mice with deficient *Trem2* gene (Trem2^−/−^) and age-matched WT mice at 12 months of age. **E** Dysfunctional microglia study in an Aβ mouse model (APPPS1): with intact *Trem2* (APPPS1 Trem2^+/+^) and deficient *Trem2* (APPPS1 Trem2^−/−^) at 12 months of age. **F** Study of mouse models at the onset of neuropathology: an Aβ mouse model (App^*NL−G−F*^), a tau mouse model (P301S), and age-matched WT mice at 2–2.5 months of age. **G** Study of mouse models with moderate neuropathology: two Aβ mouse models (App^*NL−G−F*^ and APPPS1), a tau mouse model (P301S), and age-matched WT mice at 5–6 months of age. **H** Study of a mouse model of acute ischemic stroke: mice 7 days after photothrombotic surgery (Stroke) and sham surgery (Sham) at 2 months of age. **I** Human AD continuum study: subjects with prodromal AD, AD dementia, age-matched control subjects (CTRL test), and young control subjects used for calculation of the normal synchronicity (CTRL train). *n* represents the number of subjects; the mean age is shown on the bottom right of each image

In particular, female WT (*n* = 28) (Fig. 2A), APPswe/PS2 (PS2APP, *n* = 20) (Fig. 2B), and APPswe/PS1deltaE9 (deltaE9, *n* = 16) (Fig. 2C) mice were used for pharmacological depletion of microglia by CSF1R inhibition [19] to test whether ICCs of TSPO-PET change when microglia are cleared from the brain. The mice received TSPO-PET at 6–9 (WT), 11.5 (PS2APP), or 5.5–6.5 (deltaE9) months of age and cages were randomly stratified into treatment (WT: *n* = 14; PS2APP: *n* = 10; deltaE9: *n* = 8) or vehicle (WT: *n* = 14; PS2APP: *n* = 10; deltaE9: *n* = 8) groups. CSF1R inhibition was performed by administration of PLX5622 (1200 ppm) orally in chow (Sniff Spezialdiaeten GmbH, Soest, Germany) for seven weeks (four weeks for deltaE9), and follow-up TSPO-PET scans were performed in the last week of treatment. For the WT and the PS2APP cohort, brain extraction for immunohistochemistry analyses was performed on the last day of treatment, and Iba1 staining was performed to validate microglia depletion as reported elsewhere [20].

To investigate the impact of dysfunctional microglia on microglia synchronicity, we analyzed TSPO-PET scans of female APPPS1 (APPPS1-21) mice [21] and age- and sex-matched WT with intact or deficient *Trem2* (APPPS1 Trem2^+/+^, *n* = 11; APPPS1 Trem2^−/*−*^, *n* = 11; Trem2^+/+^, *n* = 17; Trem2^−/−^, *n* = 17) at 12 months of age (Fig. 2D and E) [22]. The presence of dysfunctional microglia and Aβ pathology was validated by CD68 and X-34 staining, respectively, in [22].

For investigation of microglial synchronicity at very early pathology stages, we compared App^*NL−G−F*^ mice (APP knock-in Aβ mouse model) and P301S mice (tau mouse model) with age matched WT mice at 2–2.5 months of age using a sample size of *n* = 12 TSPO-PET scans per group (all female) (Fig. 2F).

For investigation of microglial synchronicity at stages of moderate neuropathology, we compared App^*NL−G−F*^ mice, APPPS1 mice (transgenic Aβ mouse model) and P301S mice with age-matched WT mice at 5–6 months of age using a sample size of *n* = 14 TSPO-PET scans per group (all female) (Fig. 2G). Validation of early and moderate neuropathology was performed by Aβ staining for App^*NL−G−F*^ and APPPS1 mice in [15] and by AT8 staining for P301S mice in [23].

To study the impact of acute neuroinflammation on microglia synchronicity, we compared male WT mice (*n *= 6) at 7 days after photothrombotic stroke surgery to age- and sex-matched WT mice (*n* = 6) that underwent a sham surgery (Fig. 2H) [24]. The mice were 2–2.5 months of age at the time of the surgery. Photothrombotic stroke surgery was performed as described previously [24–26]. Briefly, mice were anesthetized with isoflurane (30% O_2_, 70% N_2_O) and placed in a stereotactic frame, maintaining body temperature at 37 °C. Dexpanthenol eye ointment was applied. Mice received 10 μl/g body weight of 1% Rose Bengal (Sigma Aldrich) in saline intraperitoneally 5 min before anesthesia. After a skin incision exposed the skull, the lesion site was marked in the left hemisphere (1.5 mm lateral, 1.0 mm rostral to bregma). A 2.0 mm diameter circular area was exposed to a 25 mV laser (561 nm, Cobolt Jive 50) for 17 min, 10 min post-Rose Bengal injection. The sham procedure was performed in the same fashion, but without laser illumination. Buprenorphin and Carprofen were administered for analgesia. Post-surgery, mice were placed in a 37 °C warming chamber for 20 min.

Each PET scan contained a mixture of different animal groups, i.e., transgenic mice and WT mice, using different randomized positions per scan. In each comparison group, we used an equal number of animals per cohort to ensure comparability of the results.

### Animal PET imaging and analysis

All small animal positron emission tomography (μPET) procedures followed an established standardized protocol for radiochemistry, acquisition, and post-processing [27]. In brief, [^18^F]GE-180 TSPO μPET (TSPO-PET) with an emission window of 60–90 min post-injection was used to measure cerebral microglial activity. All analyses were performed by PMOD (V3.5, PMOD Technologies, Zurich, Switzerland) using a tracer-specific template for spatial normalization [28]. PET data were analyzed using a user-independent automatized brain normalization step to exclude operator bias in the PET data analysis. Normalization of injected activity was performed by global mean scaling, resulting in a standardized uptake value ratio (SUVR). Additionally, for each cohort, we defined a best reference region (BR) as the region that showed the lowest Cohen’s d between the transgenic/treated cohort and the corresponding WT/placebo cohort when comparing their global mean-scaled tracer uptake. Furthermore, the previously validated myocardium correction method for TSPO-PET [29] was used for validation by a brain-independent quantification method in the depletion experiment. μPET mean values were extracted from all volumes of interest (VOIs) of the Mirrione atlas [30] (excluding midbrain, inferior colliculi, hypothalamus, central grey matter, superior colliculi, and olfactory bulb), with further subdivision of the neocortical target region (visual, auditory, entorhinal, sensorimotor, and somatosensory cortex, all split into left and right) [31]. The final mouse VOI set consisted of 21 regions (9 bilateral). Cerebellum, brain stem, and right and left sensorimotor cortex VOIs were manually cropped to correct for signal spill-in from the brain ventricles and Harderian glands. All small animal PET experiments were performed with isoflurane anesthesia (1.5% at time of tracer injection and during imaging; delivery 3.5 L/min).

### Human PET imaging

34 Aβ-positive (determined by a visual Aβ-PET read) patients across the AD continuum (17 prodromal AD consisting of 5 with subjective cognitive decline (SCD) and 12 with mild cognitive impairment (MCI), 17 AD dementia) and 12 age- and sex-matched cognitively normal controls (controls, CTRL) were enrolled from the ongoing interdisciplinary and prospective AD study "Activity of Cerebral Networks, Amyloid and Microglia in Aging and AD (ActiGliA)" [4]. 13 younger controls were additionally included as a training cohort (Fig. 2I). Detailed characteristics of all included subjects are shown in Supplementary Table S2. Controls were defined as subjects without objective cognitive impairment (clinical dementia rating [CDR] global score = 0, Consortium to Establish a Registry for Alzheimer’s Disease Neuropsychological Battery [CERAD-NB] total score ≥ 69), and no indication of Aβ pathology on PET (negative visual read) and/or CSF examination (Aβ_42/40_ ratio ≥ 5.5%). All subjects were scanned at the Department of Nuclear Medicine, LMU, using a Biograph 64 PET/CT scanner (Siemens, Erlangen, Germany). The Aβ-PET status was acquired in all ActiGliA subjects using [^18^F]flutemetamol Aβ-PET. PET acquisition and PET data analyses (ethics-applications: 17–569 & 17–755) were approved by the local institutional ethics committee (LMU Munich) and the German radiation protection (BfS-application: Z 5—22,464/2017–047-K-G) authorities. All participants provided written informed consent before the PET scans. Before each PET acquisition, a low-dose CT scan was performed for attenuation correction. Emission data of TSPO-PET were acquired from 60 to 80 min [32] after the injection of 191 ± 10 MBq [^18^F]GE-180 as an intravenous bolus. The specific activity was > 1500 GBq/μmol at the end of radiosynthesis, and the injected mass was 0.13 ± 0.05 nmol. Images were reconstructed using a 3-dimensional ordered subsets expectation maximization algorithm (16 iterations, 4 subsets, 4 mm gaussian filter) with a matrix size of 336 × 336 × 109, and a voxel size of 1.018 × 1.018 × 2.027 mm^3^. Standard corrections for attenuation, scatter, decay, and random counts were applied. TSPO-PET data were preprocessed using PMOD (PMOD Technologies, Zurich, Switzerland). Spatial normalization was performed to a tracer specific template in the Montreal Neurological Institute (MNI) space analogous to mouse data processing. All images were normalized by global mean scaling and smoothed with a Gaussian filter of 6 × 6 × 6 mm^3^ to account for intersubject differences in anatomy. Additionally, similarly to the mouse study, we defined a BR for the AD cohorts as the region that showed the lowest Cohen’s d between the prodromal AD / AD dementia cohort and the CTRL cohort when comparing their global mean-scaled tracer uptake. The Schaefer 200 atlas was used for region definition. VOIs belonging to the temporal and parietal lobe were defined in this work as AD-signature network regions (94 VOIs), while VOIs belonging to the motor and sensory cortex (27 VOIs) were defined as motor-sensory network and used as control regions, since the corresponding brain areas only minor affected by AD neuropathology (see detailed description in Supplementary Table S3).

### TSPO binding status

Genotyping was performed at the Department of Psychiatry of the University Hospital LMU Munich and Regensburg. Genomic DNA was extracted from whole blood using a SQ Blood DNA kit von Omega Bio-Tek (Norcross, GA, USA) according to the manufacturer’s protocol. DNA quality was assessed by optical absorbance and gel electrophoresis. TaqMan quantitative polymerase chain reaction assays were used for amplification, Sager method for sequencing. Binding affinity of the [^18^F]GE-180 TSPO ligand is affected by the co-dominant rs6971 (Ala/Thr) single nucleotide polymorphisms (SNP) and needs to be considered in the imaging analysis [33]. Ala/Ala carriers are high-affinity binders (HAB), Thr/Thr carriers are low-affinity binders (LAB), and Ala/Thr carriers are mixed-affinity binders (MAB). Only HAB and MAB subjects were allocated for the PET-analysis, whereas *n* = 6 subjects with LAB status were excluded a priori.

### Human assessments of cognitive performance

Neurocognitive testing was performed using the mini-mental status examination (MMSE) and the clinical dementia rating (sum of boxes) (CDR SOB). All neurocognitive testing was performed by trained psychologists.

### Assessment of TSPO-PET ICC

ICC assessment was performed for all the studied groups using a modified version of the Python script reported in Grosch et al. [34]. In brief, the code calculates Pearson’s correlation [35] for each VOI pair and displays the Pearson’s R values as a 2D array, which additionally can be filtered based on the *p*- and *R*-value of the correlations.

In this work, to obtain robust ICC estimates, we additionally performed bootstrapping by resampling with replacement, i.e.:

- Each subject had an equal probability to be included in a resampled dataset,
- Each subject could be included in a resampled dataset more than once,
- The size of each resampled dataset equaled the size of the original dataset.

10,000 resampled datasets were generated for each mouse cohort and 1000 for each human cohort. For each resampled dataset, we calculated Pearson’s R for all the VOI pairs. The R values were then transformed using Fisher’s R to Z transformation [36]:

$$Z=\text{arctanh}\left(R\right)$$

to normalize the distribution of the R values obtained from the resampled datasets. The mean Z of the 10,000 resampled datasets (1000 for human data) is further referred to as ICC. ICC values > 0.5 and < -0.5 with *p* < 0.005 (mouse cohorts) and* p* < 0.001 (human cohorts) were defined as significant connections. A connection between two cortical VOIs was defined as a cortical connection, between two subcortical VOIs as a subcortical connection, between a cortical and a subcortical VOI as a cortical-subcortical connection.

### Individual assessment of microglia desynchronization

To assess microglia desynchronization in AD continuum patients relative to a cognitively normal human cohort on a single subject level, we introduced a quantitative score that we called desynchronization index (DI). DI of a single VOI was estimated as follows (Fig. 9A and Fig. 1B):

1. For 13 CTRL subjects of the young training cohort, we extracted mean [^18^F]GE-180 uptake values of all the AD-signature network VOIs.
2. Using these values, we calculated a linear fit between each VOI pair (mean of 10,000 bootstraps), representing the ICC.
3. For an individual subject to be assessed, mean [^18^F]GE-180 uptake values of the same VOIs were extracted.
4. For each VOI pair, we calculated the perpendicular distance *d* from the subject’s pair of values to the corresponding linear fit calculated in step 2.
5. The DI of VOI *i* was defined as a sum of the perpendicular distances of all the pairs with this VOI:
  $$D{I}_{i}= \sum_{j=1,j\neq i}^{N}{d}_{i,j},$$

where N is the number of VOIs.

DIs were calculated for 12 CTRL, 17 prodromal AD, and 17 AD dementia subjects. All the above-mentioned calculations were performed using a custom-made Python script (Numpy and Pandas libraries). As a control analysis, the same calculations were additionally performed using the motor-sensory network VOIs instead of the AD-signature network VOIs.

DI was then additionally calculated for all the investigated mouse cohorts. The calculation steps were identical to human, with a single difference that, to calculate the DI in the reference cohorts (listed in Supplementary Table S4), we used the leave-one-out approach: in step 1, all the subjects except for one test subject were used and in steps 3–5 the DIs were calculated for this one left-out subject; the procedure was then repeated for all the cohort subjects.

To compare the proposed DI analysis to the conventional SUVR analysis, we calculated the average SUVR and DI for each region across all subjects in both the mouse and human cohorts, followed by division of these average values by the corresponding average values in a reference cohort (Supplementary Table S4). These ratios were then compared between SUVR and DI. A higher ratio indicates a greater ability of either SUVR or DI to distinguish between normal and abnormal cohorts.

### Single-cell radiotracing

*App* ^*NL−G−F*^ (*n* = 8) and *wild-type* (*n *= 8) mice at an age of 6–7 months underwent scRadiotracing after TSPO tracer injection [37, 38]. Dedicated procedures for mouse models of neurodegenerative diseases were described previously [39] and modified as listed below. Forebrains and hindbrains were processed separately. Adult Brain Dissociation Kit (Miltenyi Biotec, 130–107-677) was used for brain dissociation according to the manufacturer's instructions. Adult mouse brains were dissected, briefly washed with PBS (Gibco), cut into small pieces, and dissociated with enzyme mix 1 and 2 using gentleMACS™ Octo Dissociator (Miltenyi Biotec, 130–096-427). The dissociated cell suspension was applied to pre-wet 70 µm cell strainer (Miltenyi Biotec, 130–110-916). The cell pellet was resuspended using cold PBS and cold debris removal solution. Cold PBS was gently overlaid on the cell suspension. After centrifugation at 3,000 × *g* and 4 °C for 10 min with acceleration at 9 and deceleration at 5, the two top phases were removed entirely. The cell pellets were collected and resuspended in 1 mL cold red blood cell removal solution followed by 10 min incubation. Cell pellets were collected for positive isolation of microglia or astrocytes, negative isolation of neurons, and proportion analyses of endothelial cells, oligodendrocytes and remaining cells (Fig. 9A). 5% of the single cell suspension were used to characterize proportions of successfully collected microglia, astrocytes, and neurons.

Microglia were isolated from the single cell suspension using CD11b MicroBeads, human and mouse (Miltenyi Biotec, 130–049-601) and a MACS separation system (Miltenyi Biotec). The prepared cell pellets were resuspended in 90 µL of D-PBS/0.5% bovine serum albumin (BSA) buffer per 10^7^ total cells. In total, 10 µL of CD11b MicroBeads per 10^7^ total cells were added and incubated for 15 min in the dark at 4 °C. Cells were washed by adding 1—2 mL of buffer per 10^7^cells and centrifuged at 300 × *g* for 5 min. The cell pellets were resuspended in 500 µL of D-PBS/0.5% BSA. The pre-wet LS columns (Miltenyi Biotec, 130–042-401) were placed onto a QuadroMACS Separator (Miltenyi Biotec, 130–090-976). The cell suspensions were applied onto the column. The columns were washed 3 times with 3 mL of D-PBS/0.5% BSA buffer. The flow-through containing the unlabelled cells was collected as the microglia-depleted fraction. The columns were removed from the magnetic field, and microglia were flushed out using 5 mL of D-PBS/0.5% BSA buffer.

Astrocytes were isolated from the CD11b(-) fraction using ACSA2 MicroBeads (Miltenyi Biotec, 130–097-678) and a MACS separation system (Miltenyi Biotec). The prepared cell pellets were resuspended in 80 µL of AstroMACS separation buffer (Miltenyi Biotec, 130–117-336) per 10^7^ total cells. In total, 10 µL of FcR blocking reagent were added and incubated for 10 min in the dark at 4 °C. Afterwards, 10 µL of anti-ACSA2 MicroBeads were added and incubated for 15 min in the dark at 4 °C. Cells were washed by adding 1 mL of AstroMACS separation buffer and centrifuged at 300 × *g* for 5 min. Cell pellets were resuspended in 500 µL of AstroMACS separation buffer. The pre-wet MS columns (Miltenyi Biotec, 130–042-201) were placed at OctoMACS Separator (Miltenyi Biotec, 130–042-109). The cell suspensions were applied onto the column, followed by washing 3 times with 500 µL of AstroMACS separation buffer. The flow-through was collected containing non-astrocytic cells as an astrocyte-depleted fraction. The columns were removed from the magnetic field, and the astrocytes were flashed out using 3 mL of AstroMACS separation buffer.

Neuronal isolation was performed in the CD11b(-)/ACSA2(-) fraction. Neuron Isolation Kit, mouse (Miltenyi Biotec, 130–115-390), was used according to the manufacturer's instructions. The prepared cell pellets were resuspended in 80 µl of PBS-0.5% BSA buffer per 10^7^ total cells. 20 μL of Non-Neuronal Cells Biotin-Antibody Cocktail was added and incubated for 5 min in the dark at 4 °C. Cells were washed and centrifuged at 300 × g for 5 min. Cell pellets were again resuspended in 80 μL of PBS-0.5% BSA buffer per 10^7^ total cells. 20 μL of Anti-Biotin MicroBeads were added and incubated for 10 min in the dark at 4 °C. The volume was adjusted to 500 µl per 10^7^ total cells with PBS-0.5% BSA buffer and then proceed to magnetic separation. The pre-wet LS columns (Miltenyi Biotec, 130–042-401) were placed at QuadroMACS™ Separator (Miltenyi Biotec, 130–090-976). The cell suspensions were applied onto the columns. The columns were washed with 2 × 1 ml PBS-0.5% BSA buffer. The flow-through containing the unlabelled cells were collected as the neuron-enriched fractions. The columns were removed from the magnetic field, and the non-neuronal cells were flushed out using 3 ml of PBS-0.5% BSA buffer.

### Gamma emission measurements

CD11b(+) microglia-enriched, ACSA2(+) astrocyte-enriched, neuron-enriched and depleted fractions were analysed to determine radioactivity of harvested cells. Radioactivity concentrations of cell pellets were measured in a high sensitive gamma counter (Hidex AMG Automatic Gamma Counter) relative to the injected activity, with decay-correction to time of tracer injection for final activity calculations.

### Flow cytometry

Flow cytometry staining was performed at 4 °C. Each microglia-enriched and astrocyte-enriched cell pellet was resuspended in 100 µL of cold D-PBS containing fluorochrome-conjugated antibodies recognizing mouse CD11b or ACSA2 (Miltenyi Biotec, 130–113-810 and 130–116-247) in a 1:50 and 1:9 dilution respectively and incubated for 10 min at 4 °C in the dark for quality control. Neuron-enriched and depleted were resuspended in 45 µl of D-PBS/0.5% BSA buffer and fluorochrome-conjugated antibodies recognizing CD90.2, CD31 and O4 (Miltenyi Biotec, 130–123-066, 130–123-813 and 130–117-711) were added in a 1:9 dilution and incubated for 10 min at 4 °C in the dark. Samples were washed with 1 mL of D-PBS and centrifuged for 5 min at 400 × *g*. Finally, cell pellets were resuspended in 500 µL of D-PBS and samples were used for flow cytometry using a MACSQuant® Analyzer. Acquired data included absolute cell numbers and purity of enriched CD11b( +) and ACSA2( +) cells in microglia/astrocyte-enriched samples as well as proportions of CD90.2 (neurons), CD31 (endothelial cells) and O4 (oligodendrocytes) for neuron-enriched and depleted samples.

### Single-cell RNA-sequencing in APP23 and WT mice

Single-cell RNA(scRNA) sequencing was performed for microglia isolated from cortex, hippocampus, and cerebellum of male C57BL/6 J (WT) and APP23 mice of 17 and 27 months of age, as previously described [40]. In brief, freshly isolated brain regions were homogenised mechanically, at 4 °C, and myeloid cells were separated using a Percoll gradient. Cells were stained with antibodies against CD45 and CD11b as well as the amyloid-binding dye Methoxy-X04 (MX04, abcam), which labels plaque-associated microglia that have taken up aggregated amyloid-β [41]. Microglia were identified as CD11b^high^/CD45^intermediate^, and plaque-associated cells as MX04^positive^ (MX04 +) by flow cytometry, using a Sony SH800Z FACS sorter. Single microglia were deposited into 384-well plates and sequenced using SmartSeq2 chemistry. After quality control, data analysis of high quality cells was performed using the R package Seurat v3 and v4 [42] and Wilcoxon rank sum test with Benjamini–Hochberg correction for pairwise statistical comparison of *TSPO* expression in microglia in the vicinity (MX04 +) and distant (MX04-) from plaques.

### Statistical analysis

A simulation analysis was conducted to analyze appropriate cohort size and data robustness for ICC analysis. A random number generator was used to reduce the cohort sizes by one mouse at a time, and a new ICC matrix was calculated for each cohort. For this analysis we used an FDG-PET cohort of P301S mice and matching WT controls. The root mean square error (RMSE) was calculated (RMSE = √(∑(ICC_P301S_ – ICC_WT_)^2^/ n_ICC_). Subsequently, the sample size-dependent effect size was calculated for individual regions using Cohen’s d and we observed robust results of ICCs at a sample size > 11 mice. Unless otherwise specified, all statistical analyses were performed using Scipy and Pingouin libraries (Python 3.7). To compare median absolute ICCs between cohorts, Wilcoxon signed-rank test (two-tailed) was used, as the underlying ICC distributions were shown to be non-Gaussian, the corresponding results are reported as Q_2_ (Q_1_, Q_3_), where Q_2_ is the second quartile, or the median, Q_1_ is the first quartile, Q_3_ is the third quartile. *p* < 0.05 was used as significance threshold. To test for differences in DI between the human cohorts, one-way analysis of variance (ANOVA) (numerator degrees of freedom = 2, denominator degrees of freedom = 43, significance threshold *p* < 0.05) and unpaired t-tests (two-tailed, significance threshold *p* < 0.05) were used. The normality of the DI distributions was assessed using Shapiro–Wilk test [43]. Based on the DIs from the VOIs where a significant difference in DI between the cohorts was reported, we performed a principal component analysis (PCA) (Scikit-learn library, Python 3.7) and tested for differences in the first principal component (PC1) between the cohorts by means of one-way ANOVA and unpaired t-tests. To assess the relationship between the DI and the MMSE and CDR SOB score, we calculated Pearson’s R and the *p*-value of the correlation. Benjamini–Hochberg false-discovery rate (FDR) adjustment [44] was applied to these *p*-values. p_FDR_ < 0.05 was used as a threshold for significant correlations. Additionally, we correlated PC1 to the above-mentioned scores. To analyze differences between SUVR and DI in discrimination of mouse transgenic/treated cohorts versus WT/placebo cohorts as well as human subjects with AD versus CTRL subjects, we performed paired t-tests (two-tailed, significance threshold *p* < 0.05). To evaluate differences in cellular TSPO tracer uptake and proportion of isolated cell fractions between WT and App^*NL−G−F*^ mice, we performed an unpaired t-test, *p* < 0.05 was used as significance threshold. Multiple regression was used to determine contributions of neurons (CD90.2), endothelial cells (CD31), oligodendrocytes (O4) and remaining cells (negative for all markers) to the radioactivity in the cell pellet.

## Results

### Microglia depletion proves the concept of microglia synchronicity assessment via TSPO-PET

**Fig. 3:**
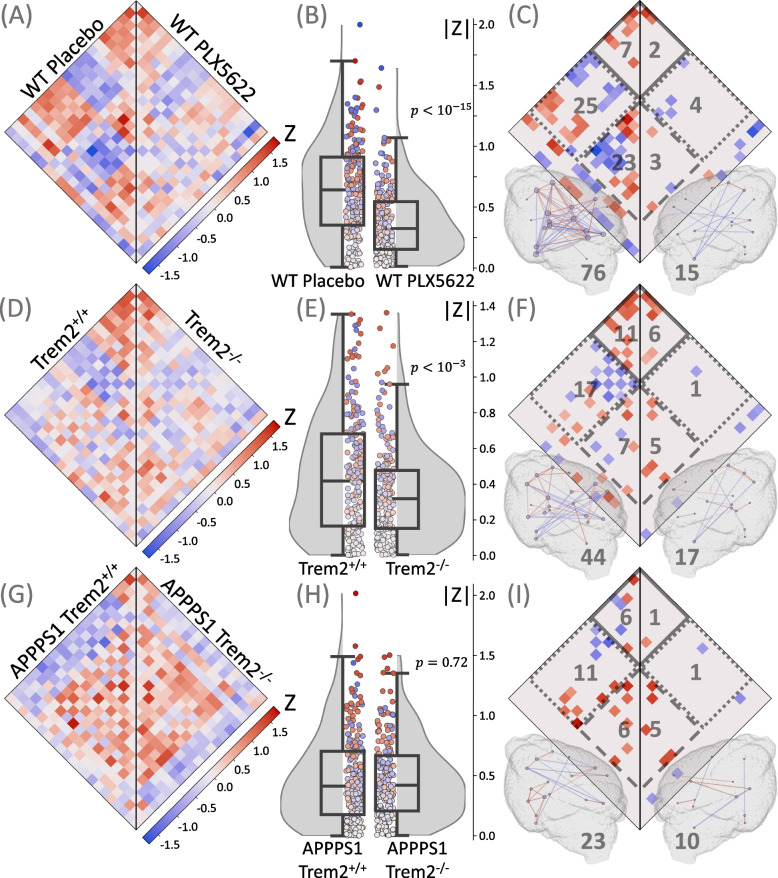
Specificity of microglia synchronicity assessment via interregional correlation coefficients (ICC). Plots show ICC heatmaps, absolute ICC values and significant connections of the depletion experiment and in mouse models with dysfunctional microglia. **A**, **B**, **C** Mice with intact microglia (WT Placebo, *n *= 14) versus mice with depleted microglia (WT PLX5622, *n *= 14). **D**, **E**, **F** Trem2^+/+^ versus Trem2^−/−^ mice. **G**, **H**, **I** APPPS1-transgenic Trem2^+/+^ versus APPPS1-transgenic Trem2^−/−^ mice. **(A**, **D**, **G)** ICC values for all the pairs of the 21 VOIs. **B**, **E**, **H** Distributions of absolute ICCs, *p*-values derive from a Wilcoxon signed-rank test. **C**, **F**, **I** Significant connections (*p* < 0.005), including cortical (solid line), subcortical (dashed line), and cortical-subcortical (dotted line) connections; the number of the corresponding connections is shown in gray. All significant connections are also projected into 3D brain images, where the color of the connection represents its value; the nodes are individual VOIs, the size of the node reflects the number of its connections; the total number of connections is shown in gray

Since PET signals are a composite of specific binding (not limited to microglia), off-target binding, and unspecific binding, we first tested whether microglia-specific synchronicity could be detected by ICCs of TSPO-PET. For this, we compared WT mice with pharmacological microglia depletion (WT PLX5622) to WT mice with intact microglia (WT Placebo) (Fig. 2A). Pharmacological depletion resulted in > 99% reduction of Iba1 positive cells in the mouse brain after seven weeks of treatment [20]. [^18^F]GE-180 uptake (SUV) was substantially reduced in the WT PLX5622 cohort compared to WT Placebo (Supplementary Fig. S1); however, it was non-zero despite the near-complete microglia depletion due to the presence of specific binding in other cell types such as endothelium and ependyma, non-specific binding, and free tracer. WT mice with intact microglia comprised a median absolute ICC of 0.642 (0.353, 0.912) (Fig. 3A and B) and a total number of 76 significant connections including 7 cortical, 23 subcortical, and 25 cortical-subcortical connections (Fig. 3C). Depleted WT mice indicated a strong reduction of the median absolute ICC (0.325 (0.154, 0.547), *p *< 10^–15^) (Fig. 3A and B) and distinct reduction of significant connections (15 total, 2 cortical, 3 subcortical, 4 cortical-subcortical connections; Fig. 3C). As a validation, we repeated the analysis with myocardium-adjusted standardized uptake values (SUVs) as an alternative normalization method and found highly consistent results (Supplementary Fig. S2). Thus, TSPO-PET data indicated the presence of a specific microglia connectome in the healthy mammalian brain.

### Microglia synchronicity is reduced in presence of dysfunctional microglia

Next, we challenged the assessment of microglia synchronicity with present but dysfunctional microglia (Fig. 2, D and E). We used mice with *Trem2* deficiency and found a pronounced reduction of the number of significant connections in both the WT and the APPPS1-transgenic model. For the WT mice (Fig. 3, D-F), *Trem2* deficiency decreased the total number of connections from 44 to 17, with the cortical-subcortical connections being affected the most (reduction from 17 to 1; Fig. 3F). The median absolute ICC dropped from 0.418 (0.165, 0.681) to 0.319 (0.152, 0.478) (*p* < 10^–3^; Fig. 3E). For APPPS1-transgenic mice with *Trem2* deficiency (Fig. 3, G-I), we observed a reduction of the total number of connections from 23 to 10 when compared to intact *Trem2*. In the Trem2^−/−^ cohort, cortical and cortical-subcortical connections were nearly absent compared to Trem2^+/+^ (6 *versus* 1 and 11 *versus* 1, respectively), while the number of subcortical connections stayed similar (6 *versus* 5) (Fig. 3I). The median absolute ICC in the APPPS1-transgenic model was not affected by *Trem2* deficiency (0.412 (0.176, 0.702) *versus* 0.420 (0.208, 0.666), *p* = 0.72) (Fig. 3H). These data indicated that dysfunctional microglia lead to reduction of microglia synchronicity, but our observations in APPPS1 mice raised the question if microglia synchronicity is already decreased in presence of Aβ, without further reduction due to *Trem2* loss of function.

### Alzheimer’s disease neuropathology decreases microglia synchronicity

Thus, we tested if alterations of microglia synchronicity can be detected early in the disease course of AD mouse models (Fig. 2F). A substantial decrease in both the median absolute ICC and the number of connections was observed in the investigated AD mouse models at both two and five-six months of age when compared to the age-matched WT mice.

**Fig. 4:**
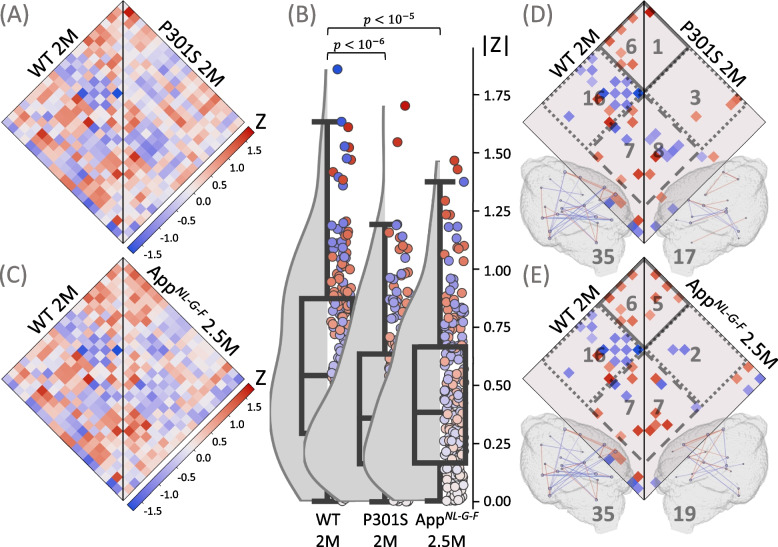
Assessment of microglia synchronicity in AD mouse models at the onset of neuropathology. Plots show ICC heatmaps, absolute ICC values and significant connections of the investigated AD mouse models at 2.0–2.5 months of age. **A**,** D** Wild-type (WT) mice (*n* = 12) versus P301S mice (*n* = 12). **C**, **E** WT mice versus App^*NL−G−F*^ mice (*n* = 12). **A**, **C** ICC values for all the pairs of the 21 VOIs. **B** Distributions of absolute ICCs, *p*-values derive from a Wilcoxon signed-rank test. **D**, **E** Significant connections (*p* < 0.005), including cortical (solid line), subcortical (dashed line), and cortical-subcortical (dotted line) connections; the number of the corresponding connections is shown in gray. All significant connections are also projected into 3D brain images, where the color of the connection represents its value; the nodes are individual VOIs, the size of the node reflects the number of its connections; the total number of connections is shown in gray

In the early cohort (2.0–2.5 months of age), the median absolute ICC dropped from 0.542 (0.294, 0.873) to 0.360 (0.163, 0.636) (*p* < 10^–6^) for P301S mice (Fig. 4A and B) and to 0.384 (0.167, 0.666) (*p* < 10^–5^) for *App* ^*NL−G−F*^ mice (Fig. 4B and C). The total number of connections was also considerably smaller in both disease models (35, 17, 19 in WT, P301S, *App* ^*NL−G−F*^, respectively), which was attributed to the reduction in cortical and cortical-subcortical connections (cortical: 6, 1, 5; cortical-subcortical: 16, 3, 2; subcortical: 7, 8, 7 in WT, P301S, *App* ^*NL−G−F*^, respectively) (Fig. 4D and E).

**Fig. 5:**
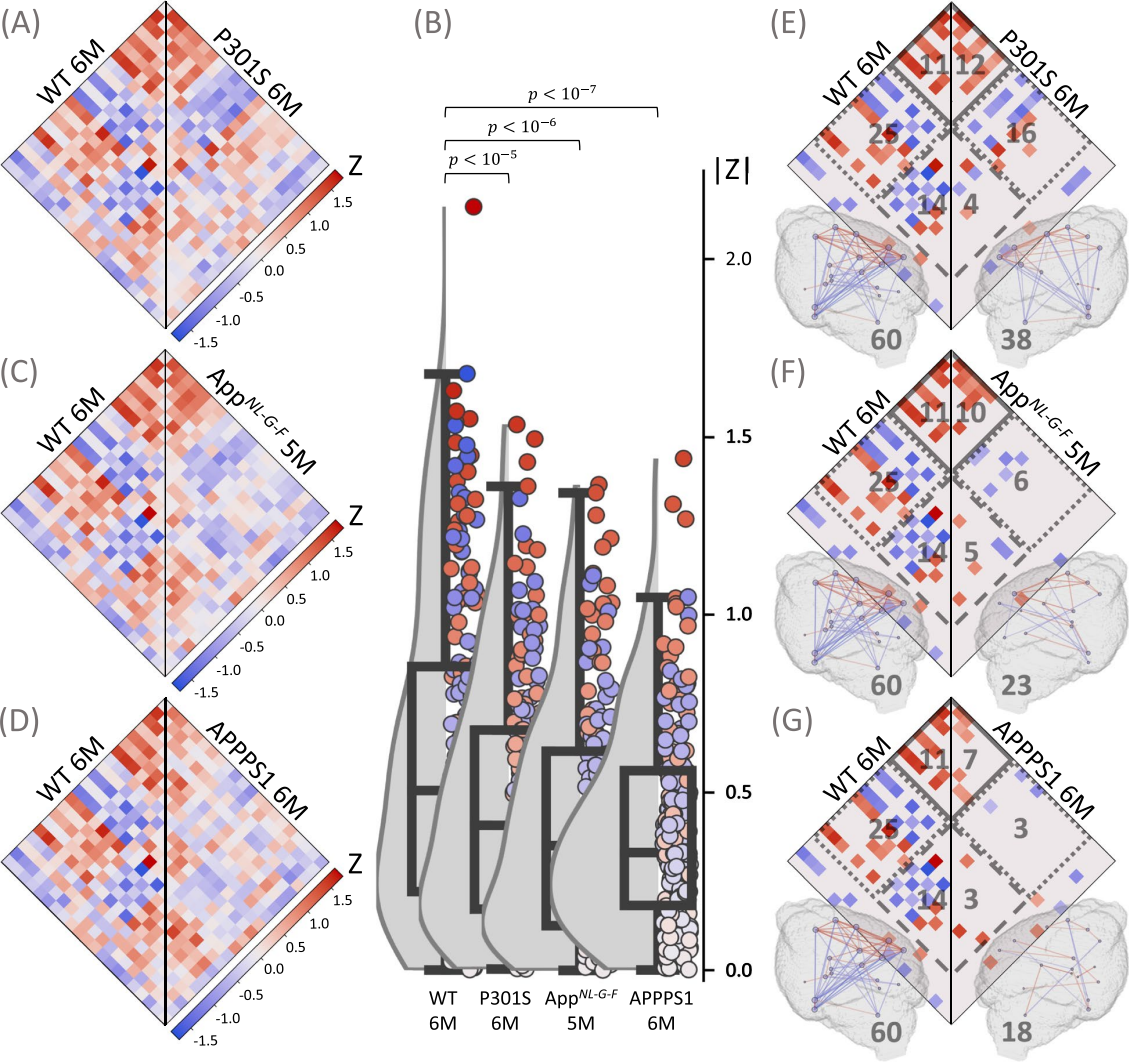
Assessment of microglia synchronicity in AD mouse models with moderate neuropathology. Plots show ICC heatmaps, absolute ICC values and significant connections of the investigated mouse models of AD at 5–6 months of age. **A**,** E** Wild-type (WT) mice versus P301S mice. **C**, **F** WT mice (*n* = 14) versus App^*NL−G−F*^ mice (*n *= 14) **D**, **G** WT mice versus APPPS1-transgenic mice (*n *= 14). **A**, **C**, **D** ICC values for all the pairs of the 21 VOIs. **B** Distributions of absolute ICCs, *p*-values derive from a Wilcoxon signed-rank test. **E**, **F**, **G** Significant connections (*p* < 0.005), including cortical (solid line), subcortical (dashed line), and cortical-subcortical (dotted line) connections; the number of the corresponding connections is shown in gray. All significant connections are also projected into 3D brain images, where the color of the connection represents its value; the nodes are individual VOIs, the size of the node reflects the number of its connections; the total number of connections is shown in gray

At five to six months of age (Fig. 5)), the median absolute ICC decreased from 0.507 (0.222, 0.856) in WT to 0.410 (0.173, 0.676) (*p* < 10^–5^) in P301S mice (Fig. 5A and B), 0.352 (0.126, 0.617) (*p* < 10^–6^) in *App* ^*NL−G−F*^ mice (Fig. 5B and C), and 0.333 (0.184, 0.562) (*p* < 10^–7^) in APPPS1-transgenic mice (Fig. 5B and D). The reduction in the total number of connections (from 60 to 38, 23, and 18 in P301S, *App* ^*NL−G−F*^ mice, APPPS1-transgenic, respectively) was mainly associated with the loss in subcortical and cortical-subcortical connections (subcortical: 14, 4, 5, 3; cortical-subcortical: 25, 16, 6, 3; cortical: 11, 12, 10, 7 in P301S, *App* ^*NL−G−F*^ mice, APPPS1-transgenic, respectively) (Fig. 5E–G). In summary, microglia synchronicity was already decreased at earliest stages of neuropathological changes, where conventional TSPO-PET read-outs are insensitive to detection of microglial activation [45, 46].

### Microglia depletion in AD mouse models aggravates desynchronization

**Fig. 6:**
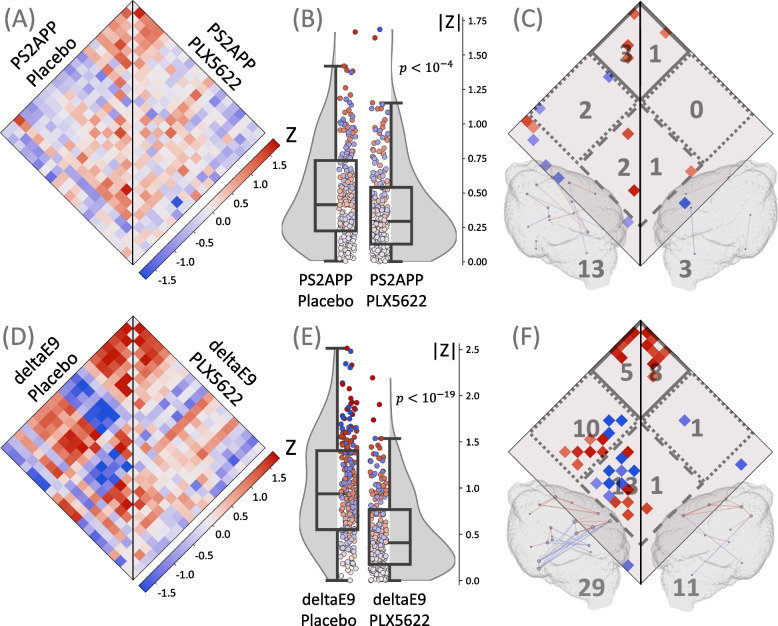
Assessment of microglia synchronicity in Aβ mouse models after microglia depletion. Plots show ICC heatmaps, absolute ICC values and significant connections of the investigated Aβ mouse models. **A**, **B**, **C** PS2APP mice with intact microglia (Placebo, *n *= 10) versus PS2APP mice with depleted microglia (PLX5622, *n *= 10). **D**, **E**, **F** DeltaE9 mice with intact microglia (Placebo, *n *= 8) versus deltaE9 mice with depleted microglia (PLX5622, *n *= 8). **A**, **D** ICC values for all the pairs of the 21 VOIs. **B**, **E** Distributions of absolute ICCs, *p*-values derive from a Wilcoxon signed-rank test. **C**, **F** Significant connections (*p* < 0.005), including cortical (solid line), subcortical (dashed line), and cortical-subcortical (dotted line) connections; the number of the corresponding connections is shown in gray. All significant connections are also projected into 3D brain images, where the color of the connection represents its value; the nodes are individual VOIs, the size of the node reflects the number of its connections; the total number of connections is shown in gray

To prove that the remaining TSPO-PET synchronicity in AD mouse models is also attributed to microglia, we performed microglia depletion with PLX5622 in AD mice and compared them to AD mice with intact microglia (placebo treatment). Two Aβ mouse models were tested: APPswe/PS2 (PS2APP) and APPswe/PS1deltaE9 (deltaE9) (Fig. 6). Brains of PS2APP mice were exemplified analyzed via immunohistochemistry and showed 66% reduction of Iba1-positive cells after seven weeks of treatment, as reported in our previous publication [20]. Similar to WT mice, we observed a reduction in both the median absolute ICC and the number of connections; however, as expected by incomplete depletion, the effect was not as strong as in WT mice.

In the PS2APP cohort, the median absolute ICC was reduced from 0.415 (0.225, 0.735) in mice after placebo treatment to 0.294 (0.129, 0.541) in mice after PLX5622 treatment (*p* < 10^–4^) (Fig. 6A and B) and the total number of connections dropped from 13 to 3 (cortical: from 3 to 1; subcortical: from 2 to 1; cortical-subcortical: from 2 to 0) (Fig. 6C). In the deltaE9 cohort, the median absolute ICC reduction in PLX5622-treated animals was more prominent: from 0.937 (0.550, 1.405) to 0.410 (0.175, 0.767) (Fig. 6D and E). A substantial decline in the number of connections in deltaE9 mice was attributed to the loss in subcortical and cortical-subcortical connections (cortical: 5, 8; subcortical: 13, 1; cortical-subcortical: 10, 1 in placebo- and PLX5622-treated mice, respectively) (Fig. 6F).

### Mouse cohorts show reduced microglial synchronicity, with cortical-subcortical connections being most affected

**Fig. 7:**
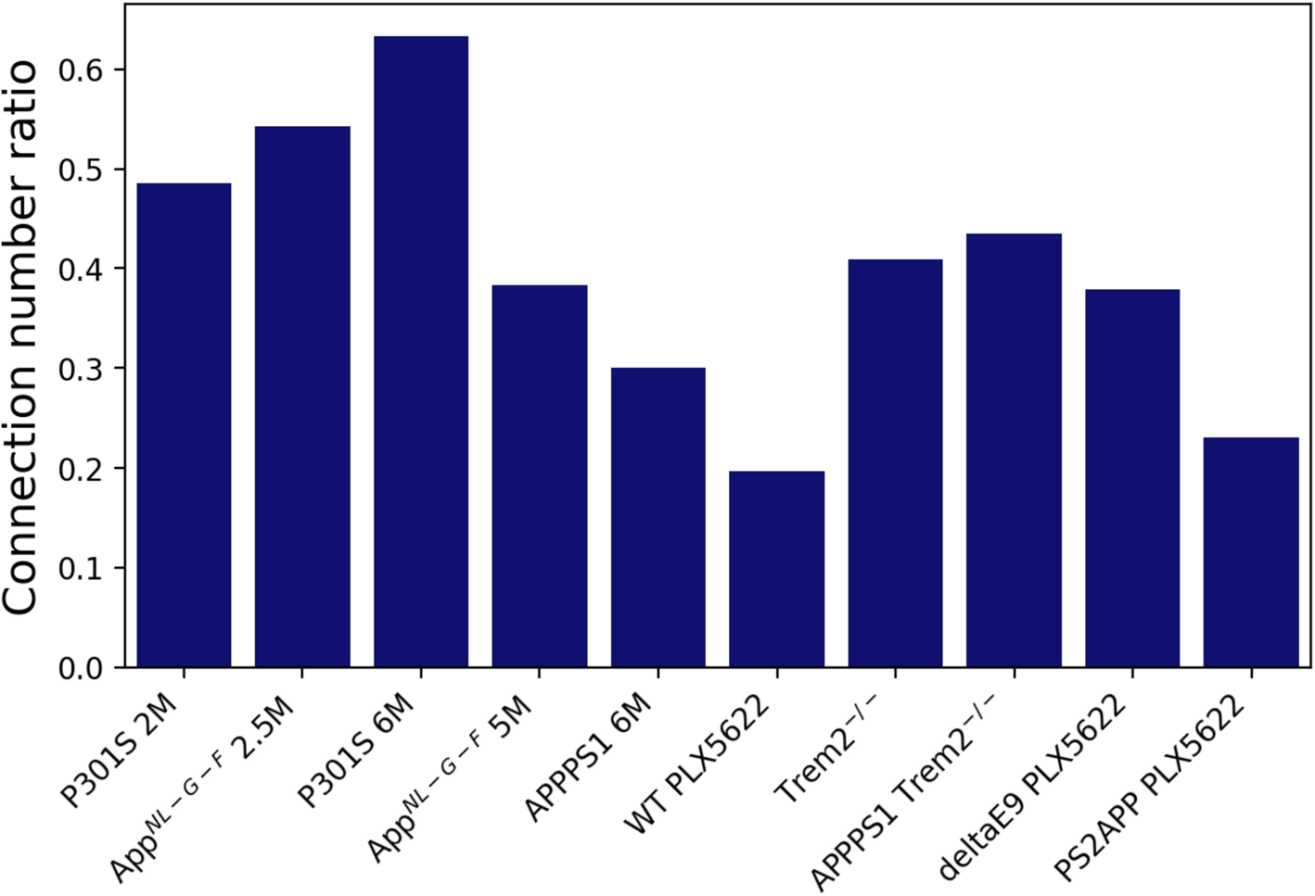
Ratio of the total number of connections in the investigated mouse cohorts relative to their corresponding reference cohorts. The name of each reference cohort can be found in Supplementary Table S4

Since the exact number of connections is subject to the number of subjects in the cohort and various batch effects, we standardized the results to enable comparison between cohorts from separate experiments. For this, we divided the number of connections in each cohort by those in the corresponding reference cohort, yielding the connection number ratio for all connections (Fig. 7), cortical connections (Supplementary Fig. S3A), cortical-subcortical connections (Supplementary Fig. S3B), and subcortical connections (Supplementary Fig. S3C). However, caution is needed when comparing cohorts with non-equivalent reference cohorts (non-WT reference cohorts were used for APPPS1 Trem2^−/−^, deltaE9 PLX5622, and PS2APP PLX5622). The most prominent reduction in the connection number ratio in most cohorts occurred between the cortex and the subcortex (Supplementary Fig. S3B). Figure 7 Notably, the WT cohort with depleted microglia (WT PLX5622) showed the lowest total connection number ratio, underscoring the microglial specificity of the observed desynchronization. Similarly, deltaE9 PLX5622 and PS2APP PLX5622 cohorts showed a reduced total connection number; however, not as low as WT PLX5622, likely due to incomplete depletion and already reduced synchronicity in their reference cohorts (deltaE9 Placebo and PS2APP Placebo, respectively) due to neuropathology. All the three PLX5622 cohorts demonstrated prominent reductions in the number of connections in cortical-subcortical (Supplementary Fig. S3B) and subcortical (Supplementary Fig. S3C) networks, with WT PLX5622 and PS2APP PLX5622 also showing reductions in the cortical network. Comparing AD mouse models at the onset of neuropathology to those with moderate neuropathology reveals that the younger Aβ mouse model (App^*NL−G−F*^ 2.5 M) had a higher total connection number ratio (0.54) than the older models (App^*NL−G−F*^ 5 M and APPPS1 6 M; 0.38 and 0.30, respectively) (Fig. 7). In contrast, the tau model (P301S) showed an increase from 0.49 at 2 M to 0.63 at 6 M (Fig. 7). Nevertheless, all AD mouse models demonstrated a three to fourfold reduction in the subcortical connection number ratio at moderate neuropathology compared to the onset (Supplementary Fig. S3C).

Both Trem2^−/−^ and APPPS1 Trem2^−/−^ showed over a 50% reduction in the total number of connections compared to their respective reference cohorts (Trem2^+/+^ and APPPS1 Trem2^+/+^) (Fig. 7). The impact of TREM2 deficiency on cortical microglia synchronicity was more pronounced in the APPPS1-transgenic mice (Supplementary Fig. S3A, cortical connection number ratio 0.17 versus 0.55 in Trem2^−/−^). The effect on subcortical connections and connections between the cortex and the subcortex was similar in both models.

### Translation of microglia synchronicity assessment to human TSPO-PET recapitulates preclinical findings

**Fig. 8:**
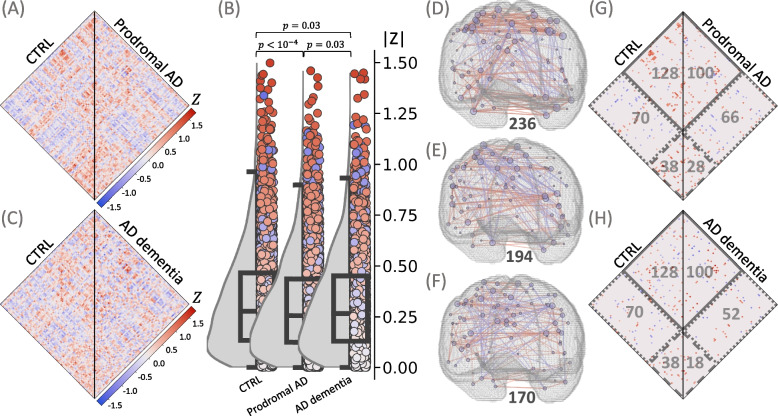
Assessment of microglia synchronicity in patients of the AD continuum and healthy controls. **A**, **G** Cognitively normal subjects (CTRL, *n* = 17) versus subjects with subjective memory decline or mild cognitive impairment (prodromal AD, *n* = 17). **C**, **H** Cognitively normal subjects (CTRL) versus subjects with AD dementia (*n* = 17). **A**, **C** ICC values (Fisher’s Z) for all the pairs between the 94 VOIs. **B** Distributions of absolute ICCs, *p*-values derive from a Wilcoxon signed-rank test. **D**, **E**, **F** Significant connections of CTRL, prodromal AD, and AD dementia, respectively, projected into 3D brain images. The color of the connection represents its value; the nodes are individual VOIs, the size of the node reflects the number of its connections; the total number of connections is shown in black. **G**, **H** Significant connections, including parietal (solid line), temporal (dashed line), and temporal-parietal (dotted line) connections; the number of corresponding connections is shown in gray. CTRL = healthy control; AD = Alzheimer’s disease

We applied the same approach for TSPO-PET ICCs to human data including healthy controls and patients of the AD continuum (Supplementary Table S2), yielding similar results as compared to the preclinical study. To achieve an equal number of subjects per cohort and thus comparability, here the healthy control cohort (CTRL) consisted of 17 subjects: 13 CTRL (train) and 4 CTRL (test). The median absolute ICC of the CTRL cohort was significantly higher when compared to patients with prodromal AD and AD dementia (0.276 (0.133, 0.466) *versus* 0.256 (0.124, 0.436); *p*_*CTRL-prodromal*_ < 10^–4^) and 0.266 (0.129, 0.451); *p*_*CTRL-ADD*_ = 0.03), respectively, Fig. 8A-C). The median absolute ICC of the prodromal AD cohort was slightly lower when compared to the AD dementia cohort (*p* = 0.03, Fig. 8B). A stage-dependent decrease was demonstrated for the total number of significant connections (CTRL: 236, prodromal AD: 194, AD dementia: 170, Fig. 8D-F). The number of parietal connections dropped from 128 in CTRL to 100 in both prodromal AD and AD dementia. The number of parietal-temporal connections decreased from 70 in CTRL to 66 in prodromal AD and 52 in AD dementia. The most prominent reduction was demonstrated for temporal connections (CTRL: 38, prodromal AD: 28, AD dementia: 18) (Fig. 8G and H).

### A personalized microglia desynchronization index of AD signature regions shows stage-dependent progression in the AD-continuum and association with cognitive performance

**Fig. 9:**
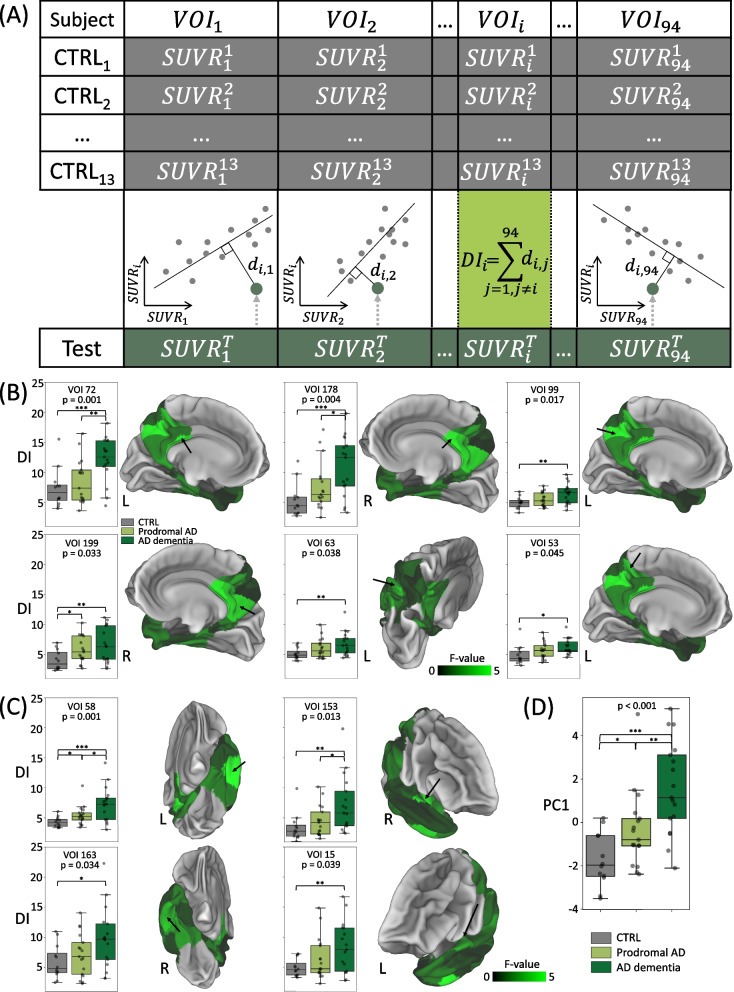
Personalized desynchronization of microglia connectome in patients of AD continuum when compared to healthy controls. **A** Calculation of the desynchronization index (DI) for an exemplary VOI_i_ $\mathrm{SUVR}_{\mathrm i}^{\mathrm j}$ is mean SUV ratio in VOI i of patient j. Mock data are shown. **B**, **C** Individual DIs calculated for cognitively normal (*n*=12) and patients of the AD continuum (prodromal AD: *n*=17, AD dementia: *n*=17). Only VOIs with significant differences in DI are shown. **B** Parietal VOIs, **C** Temporal VOIs. **D** First principal component (PC1) calculated based on the VOIs with significant differences in DI. Each dot represents an individual subject. Unpaired t-test: * - *p* < 0.05, ** - *p* < 0.01, *** - *p* < 0.0001. The *p*-value of one-way ANOVA is shown on top of each plot. Each VOI is shown on a 3D surface with a black arrow, R and L represent the hemisphere where the VOI is located. The 3D surfaces display F-values (one-way ANOVA) for all the 94 temporal and parietal Schaefer VOIs

Next, we developed an index of individual regional desynchronization of microglial activation in controls and patients of the AD-continuum (Fig. 9A). For the AD-signature network, we were able to demonstrate that the individual desynchronization index increases with the stage of the AD-continuum. Significant differences in the desynchronization index between CTRL, prodromal AD, and AD dementia were observed in six parietal (Schaefer 72, 178, 99, 199, 63, 53, Fig. 9B) and four temporal (Schaefer 58, 153, 163, 15, Fig. 9C) VOIs. The left hemisphere was slightly more affected (six VOIs with significant differences in desynchronization index of the left hemisphere *vs.* four VOIs in the right hemisphere). Contrary, the motor-sensory network did not show any stage-dependent increases of the desynchronization index, but a slight decrease in two regions of interest (Supplementary Fig. S4). To account for multiple comparisons and to generate a single read-out per subject, we applied a data-driven approach and compared the principal component of the desynchronization index (derived from the ten top-rated VOIs above) between CTRL, prodromal AD, and AD dementia. Here, we found high differences of principal component 1 across groups (*p* < 0.001) and strong discrimination for all group-wise comparisons (Fig. 9D).

### The personalized microglia desynchronization index demonstrates higher discriminative value compared to the conventional SUVR analysis in both human and mouse cohorts

We translated our DI methodology from the human cohorts back into the mouse cohorts to validate the clinical value of the DI compared to the SUVR analysis (Supplementary Fig. S5). DI-based analysis demonstrated a significantly greater ability to distinguish between normal and abnormal cohorts compared to SUVR-based analysis in most of the mouse cohorts (7 out of 10, exceptions were PS2APP PLX5622, Trem2^−/−^, and P301S 2 M) and all the human cohorts with large effect (Cohen’s d > 0.8). BR scaling further improved the discriminative value of the DI, in this case only one cohort (P301S 2 M) showed no difference between the DI and the SUVR.

**Fig. 10:**
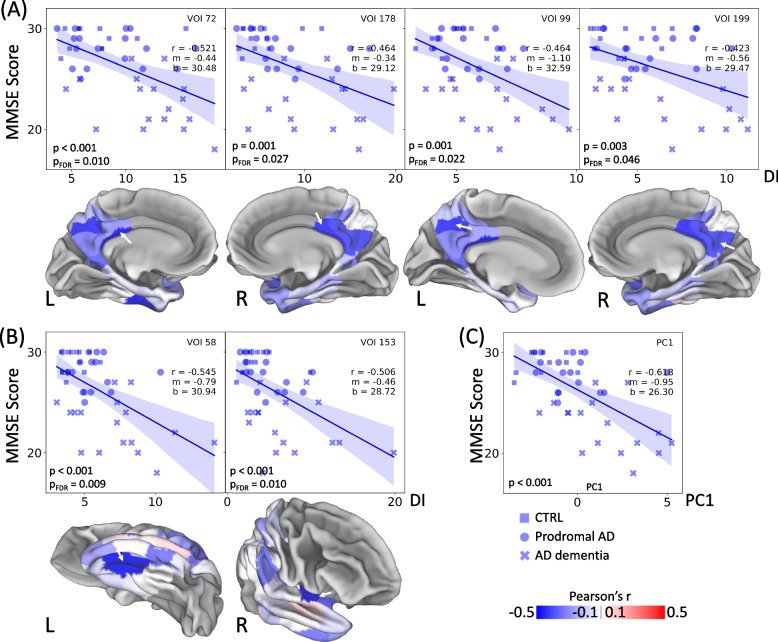
Personalized desynchronization of microglia connectome correlates with cognitive performance. Relationships between the desynchronization index (DI) and MMSE scores are shown for VOIs with significant correlations after FDR correction. **A** Parietal VOIs, (**B**) temporal VOIs. p is the correlation *p*-value, p_FDR_ is the corresponding *p*-value after FDR correction, r is the Pearson’s r, m is the slope of the fit, b is its intercept. 3D surfaces display Pearson’s r for all the 94 temporal and parietal Schaefer VOIs. The localization of the VOIs is shown by the white arrow on the corresponding 3D brain surface. R and L represent the hemisphere of the VOI. **C** Relationship between the first principal component (PC1), calculated based on the VOIs with significant differences in DI compared to CTRL (Fig. 9), and MMSE scores. CTRL: *n*=12, prodromal AD: *n*=17, AD dementia: *n*=17

Furthermore, the individual desynchronization index was negatively correlated with the MMSE score in the investigated patients of the AD-continuum. Significant negative correlations after FDR-correction for multiple comparisons were observed in four parietal (Schaefer 72, 99, 178, 199, Fig. 10A and Supplementary Table S5) and two temporal (Schaefer 58, 153, Fig. 10B and Supplementary Table S5) VOIs. Additionally, a positive correlation between the desynchronization index and the CDR SOB score was obtained for 22 parietal (Schaefer 99, 63, 72, 81, 25, 44, 149, 178, 24, 46, 199, 35, 96, 36, 19, 62, 17, 61, 34, 53, 27, 141) and 8 temporal (Schaefer 58, 15, 153, 60, 164, 152, 163, 100) VOIs (Supplementary Fig. S6 and Supplementary Table S6). The first principal component of the desynchronization index was strongly correlated with MMSE (*r* = -0.618, *p* < 0.001, Fig. 10C) and CDR SOB (*r* = 0.681,* p* < 0.001, Supplementary Fig. S6). In summary, the personalized desynchronization index indicated potential to serve for AD stage-dependent assessment of the microglia connectome.

### Single-cell radiotracing confirms microglia as the driver of regionally desynchronized translocator protein radiotracer binding in presence of amyloidosis

**Fig. 11:**
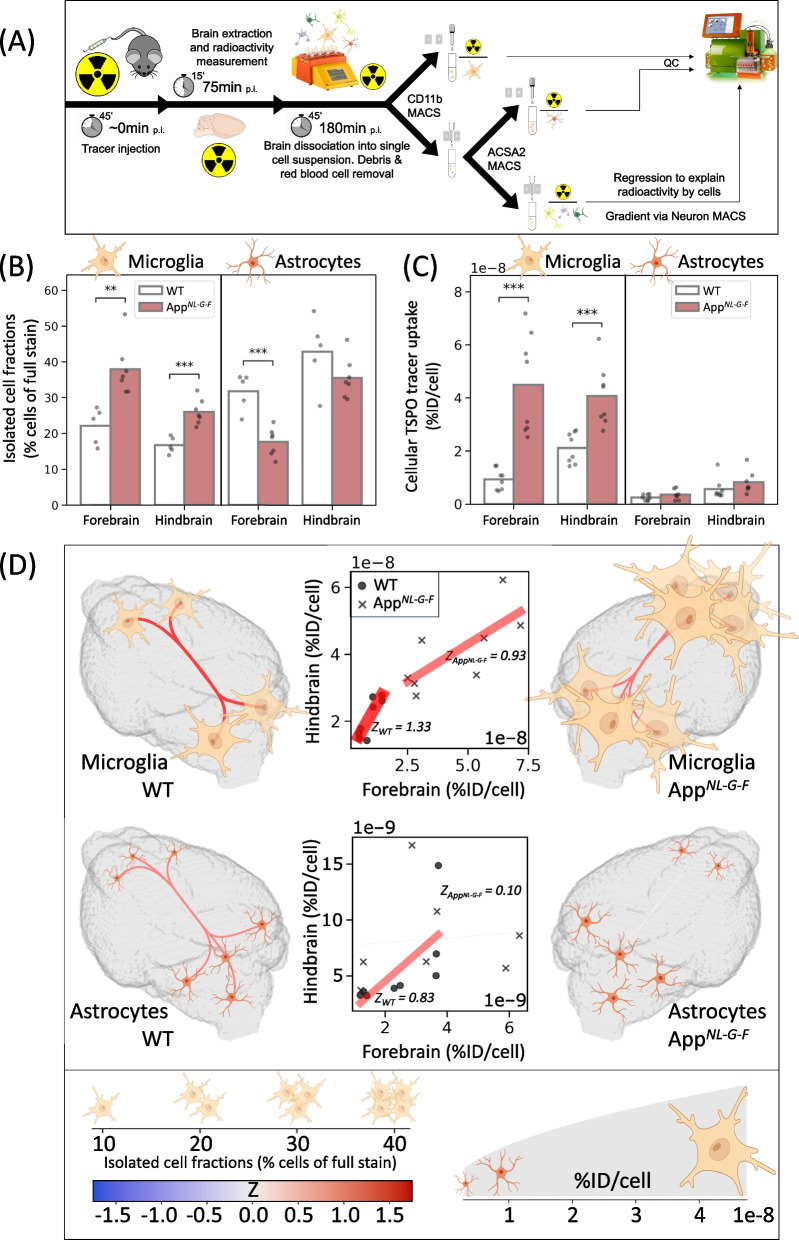
Single-cell radiotracing disentangles microglia as biological driver of TSPO-PET desynchronization in an AD mouse. **A** scRadiotracing workflow. **B** Percentage of isolated microglia and astrocytes fraction in the single cell suspension of WT (*n*=5) and App^*NL-G-F*^ mice (*n*=7). **C** Cellular TSPO tracer uptake in microglia and astrocytes as identified by scRadiotracing (WT: *n*=8, App^*NL-G-F*^: *n*=8). Unpaired t-test: **: *p* < 0.01, ***: *p* < 0.001. Each point corresponds to a single mouse. **D** Graphical summary of (**B**) and (**C**): the number of cells in 3D brain images is proportional to the isolated cell fraction in the corresponding region; the size of the cells is related to the cellular TSPO tracer uptake in the region (square root relationship for display purpose). The scatter plots display the relationship between the cellular tracer uptake in the forebrain and the hindbrain; each point stands for a single animal; the straight lines represent linear fits calculated per cohort. Line color and thickness are proportional to Fisher’s Z and absolute Fisher’s Z, respectively, both in scatter plots and in 3D representations. Lower absolute Fisher’s Z corresponds to a higher degree of forebrain-hindbrain desynchronization. Estimated contributions of non-microglia/non-astrocyte cell types and gating strategies of quality control are presented in Supplementary Fig. S7

To investigate the biological basis of regional (de)synchronization and the cellular sources of TSPO radiotracer binding, we performed scRadiotracing on WT and App^*NL−G−F*^ mice after TSPO tracer injection (Fig. 11A) [38, 47]. Since most prominent changes of synchronicity appeared between cortical and subcortical regions (Supplementary Fig. S3B), we evaluated the cellular TSPO radiotracer uptake separately in forebrain and hindbrain regions. The relative proportion of microglia in the cellular yield was significantly increased in App^*NL−G−F*^ compared to WT (forebrain: *p* < 0.01, hindbrain: *p* < 0.001), while the relative proportion of isolated astrocytes was reduced in forebrain (*p* < 0.001) (Fig. 11B). App^*NL−G−F*^ mice demonstrated enhanced microglial TSPO tracer uptake compared to WT (*p* < 0.001), whereas no differences in astrocyte uptake were detected between genotypes (Fig. 11C). Importantly, microglial TSPO tracer uptake strongly exceeded TSPO tracer uptake of astrocytes in WT and App^*NL−G−F*^ mice, identifying microglia as the main cellular source of regionally connected PET signals in the mouse brain. A regression analysis identified endothelial cells as another relevant source of our TSPO-PET signals in the mouse brain, whereas neurons, oligodendrocytes and remaining cells only showed minimal tracer uptake, regardless of genotype and brain area (Supplementary Fig. S7). Building upon the variably elevated microglial tracer uptake per cell, microglia showed a reduced correlation of the TSPO uptake between forebrain and hindbrain in presence of amyloidosis (*Z* = 1.33 in WT *versus Z* = 0.93 in App^*NL−G−F*^) and thus reduced forebrain-hindbrain synchronicity (Fig. 11D). The correlation of astrocyte TSPO tracer uptake between forebrain and hindbrain was also strongly reduced in presence of amyloidosis (*Z* = 0.83 in WT *versus Z* = 0.10 in App^*NL−G−F*^). Finally, we analyzed *TSPO* expression in a scRNA dataset of APP23 and WT mice and found enhanced *TSPO* in the cortex and the hippocampus but not in the cerebellum of aged mice with amyloidosis (Supplementary Fig. S8A). Moreover, *TSPO* expression was highest in plaque-associated microglia, as identified by methoxy-04-positivity (Supplementary Fig. S8, B and C). In summary, the region-specific increase of variable *TSPO* expression and TSPO tracer uptake in microglia was identified as the dominant biological source of desynchronized PET imaging patterns.

### Acute neuroinflammation by photothrombotic stroke surgery is associated with microglia desynchronization

Finally, we questioned whether microglia desynchronization observed in chronic neuroinflammation also occurs in acute neuroinflammation. We investigated microglia synchronicity in a photothrombotic stroke mouse model seven days post-surgery and compared it to sham mice (Supplementary Fig. S9). Similarly to the AD mouse models, the stroke mice showed pronounced desynchronization, with median absolute ICC decreasing from 0.770 (0.363, 1.301) to 0.539 (0.241, 0.881). The number of connections dropped from 26 to 7, with cortical-subcortical connections being most affected (reduced from 12 to 0).

## Discussion

This translational study integrates preclinical proof of concept in mouse models of Aβ and tau pathology together with clinical neuroimaging data to provide evidence for microglia synchronicity that can be assessed in vivo by TSPO-PET. Microglia indicated synchronicity in WT mice, which was strongly decreased upon near-to-total microglia depletion. Data from AD mouse models indicate loss of synchronicity in the presence of dysfunctional microglia as well as in presence of β-amyloid and tau pathology, even at stages of neuropathology onset. Residual synchronicity in Aβ mouse models was further reduced upon microglia reduction, thus demonstrating microglial origin of TSPO-PET synchronicity. Stage-dependent decrease of microglia synchronicity measured by DI and its strong association with cognitive performance indicates potential as a personalized biomarker of the microglia connectome in AD. Finally, cell sorting after in vivo radiotracer injection in mice determined higher variability of TSPO tracer uptake in disease-associated microglia as the biological source of regional desynchronization.

Since regional PET tracer uptake will inter-correlate to a certain degree in presence of any regional target heterogeneity, specific binding in endothelial cells and ependyma, off-target binding, or unspecific background signal, we endeavored to prove specificity of the proposed microglia connectome, which constitutes the key finding of this work. To this end, we made use of pharmacological depletion of microglia by CSF1R inhibition using PLX5622 that removed 99% of all Iba1-positive microglia cells at the time of the TSPO-PET scan. Strikingly, the regional ICCs of TSPO-PET in the healthy mouse brain dropped dramatically upon this near complete depletion of microglia, confirming the specificity of regionally inter-correlated TSPO-PET to synchronized microglial activation. CSF1R inhibition in amyloid mouse models also reduced ICCs of TSPO-PET compared to mice with amyloidosis and intact microglia, although depletion of microglia was incomplete (66%) after seven weeks of treatment. In our previous publication [48], in a tau mouse model (P301S), we achieved > 90% depletion using CSF1R inhibition. Thus, the remaining regional TSPO-PET synchronicity in AD mouse models can also be attributed to microglia. We note that diphtheria toxin or receptor-based strategies could remove microglia in amyloid mouse models more effectively, which would allow studying the cellular specificity of TSPO-PET synchronization in AD mouse models in more detail.

One interesting question that arises from this finding is the underlying mechanism of synchronized microglia activation in the healthy mammalian brain. First, the normalization methods of TSPO-PET deserve discussion. We applied global mean normalization and the strong decrease of ICCs upon microglia depletion was highly consistent with myocardium-adjusted SUV scaling as an alternative normalization method [29]. Global mean scaling minimized confounds that could be introduced by high inter-animal variability of *TSPO* expression as a driver of regional synchronicity. As an example, high variability of global *TSPO* expression would likely result in high ICCs, even if *TSPO* expression is not regionally dependent. Mediators of microglial activation could specifically drive the innate immune system of the brain in a synchronized fashion. Although the healthy brain is dominantly characterized by a homeostatic microglia signature [49], maintenance functions of microglia such as synaptic pruning [50] or debris removal [51] require activation of microglia. We find some evidence for this hypothesis since microglia synchronicity decreased in mice with *Trem2* loss of function that locks microglia in a homeostatic state [52]. Thus, at least parts of the observed microglia connectome depend on mediators of microglia activation. Decreases of microglia synchronicity in presence of Aβ and tau pathology also fit into this hypothesis since predilection sites of protein aggregation are also prone to alterations in microglia signaling [53]. Nevertheless, we believe that DAM in amyloid-rich regions is also synchronized to a certain degree. This would explain the observed microglia desynchronization in the APPPS1 *Trem2* ^−/−^ model compared to the APPPS1 *Trem2* ^+/+^ model, where *Trem2* knock-out prevents the formation of DAM. Noteworthy, a recent study even linked *TREM2* risk variants with atypical topology and phenotype of AD [54], which further supports the concept of regional microglia desynchronization upon alteration of *TREM2* function. Based on these observations, we performed cell sorting after radiotracer injection [38] to deepen the understanding of the biological sources of regional desynchronization in TSPO tracer uptake. Using analysis of brain regions with high (forebrain) and low (hindbrain) amyloidosis in *App* ^*NL−G−F*^ mice compared to WT, microglia were identified as the dominant cellular contributor of TSPO-PET signals. More variable TSPO tracer uptake was observed in microglia of amyloid-rich brain regions and scRNA confirmed that highest *TSPO* expression occurred in plaque-associated microglia. Thus, as a logical consequence, microglia cells showed lower interregional correlation of TSPO tracer uptake in presence of amyloid pathology, which explained the PET derived findings at the cellular level. Future studies could also use spatial transcriptomics [55] to investigate regional synchronicity of microglia gene expression profiles in healthy and disease states.

The second major finding of our work was very early alterations of microglia synchronicity in commonly used mouse models of AD neuropathology. Neither *App* ^*NL−G−F*^ nor P301S mice showed changes of commonly used TSPO-PET ratio values at 2.0 and 2.5 months of age [45, 46] but already strong decreases of microglia synchronicity. This finding was reproduced in *App* ^*NL−G−F*^ and P301S cohorts at ages of low to moderate neuropathology, indicating that microglia connectivity could serve as a sensitive biomarker which detects early alterations of the innate immune response in AD. We challenged the observation of early alterations in microglia synchronicity by human data and found significant desynchronization of posterior cingulate cortex and limbic temporal pole connections already at the clinical stage of SCD and MCI. In line, very early metabolic changes in AD were also found in the posterior cingulate cortex [56] and the posterior cingulate cortex synchronized degeneration network predicted progression of AD [57]. In addition, our study showed that microglial desynchronization in posterior brain regions is not only an early feature in the disease course, but it is also significantly related to worse cognitive performance in patients on the AD continuum. This confirms previous studies showing a detrimental effect of severe microglial activation on clinical severity [58–63] and cognitive decline over time [64, 65]. In this context, an index of microglia desynchronization may facilitate sensitive assessment and monitoring of early changes in microglia response to AD neuropathology also in human individuals, and ultimately patient stratification in future clinical trials [66]. Accordingly, our data build the basis to further investigate microglia synchronicity as a standardized index of disease stage dependent alterations of the innate immune response in the mammalian brain. The proposed individual DI could help to overcome lacking standardization of TSPO-PET across centers, enabling multicenter studies and head-to-head comparisons of different TSPO tracers. Moreover, the DI was proven to have a higher discriminative value compared to the conventional SUVR analysis in most of the investigated cohorts. Our data should also be considered in the context of recent studies reporting on associations of microglial activation with functional and structural brain connectivity. The consistent finding of these studies was a decrease in functional connectivity when PET detected high levels of *TSPO* expression in the brain [4, 67, 68].

Our study has limitations. First, we note that TSPO is not entirely specific to microglia [69], which may explain residual significant regional connections after microglia depletion that could result from regional similarities of reactive astrocytes or endothelial cells. In this regard, modulation of reactive astrogliosis [70] could facilitate investigation of the impact of astrocytes on TSPO synchronicity in future studies. Second, we acknowledge the relatively small sample size of human PET cohorts. The difference in the median absolute ICC between the human cohorts is small, even though statistically significant. However, the subjects with prodromal AD and AD dementia demonstrated 18% and 26% reduction, respectively, in the number of significant connections compared to CTRL, which supports the hypothesis of the stage-dependent desynchronization. The replication of these findings with larger and multicenter clinical cohorts will validate the generalizability of our results. Apart from the acute neuroinflammation study and the cohorts used for the scRNA sequencing, the preclinical studies were performed only in female mice that may show stronger differences of TSPO in Aβ mouse models compared to male mice [71]. An investigation of sex differences in microglia synchronization will be a topic of our future work. Furthermore, this study demonstrated that the magnitude and pattern of observed microglia desynchronization varied across the investigated AD mouse models, indicating a model-dependent effect. Therefore, the conclusions of this study can only be applied with caution to other models not examined in this research. The sample size in the mouse cohorts was chosen based on the results of a dedicated robustness study of metabolic connectivity in AD mouse models, showing that accurate and robust connectivity estimation is possible starting from sample sizes ≥ 12. Therefore, the results obtained for the deltaE9 and photothrombotic stroke studies should be considered preliminary and interpreted with caution, also since the degree of microglia depletion was not verified by immunohistochemistry in deltaE9 mice. Notably, in the photothrombotic stroke mouse model, acute neuroinflammation is primarily confined to the stroke region. Factors such as variability in mouse anatomy, the precision of light delivery, and the distribution of the photosensitizing agent can influence the area affected by the neuroinflammation. Studying microglia synchronicity in mice with lipopolysaccharide-induced neuroinflammation, which affects the entire brain, could provide further insights into microglial desynchronization during acute neuroinflammation.

Additionally, while the use of ICCs has shown promise, interpreting them remains challenging. Unlike the DI, ICCs can only be calculated at the cohort level. Further studies are needed to clarify the meaning of ICCs for individual region pairs. Thus, for assessing microglia desynchronization in individual subjects, we recommend using the DI.

We standardized ICCs by scaling the number of connections in each cohort to those in the respective reference cohort. However, this standardization has limitations: the reference cohorts cannot be directly compared to each other or to other cohorts, and comparisons involving the cohorts with non-equivalent reference cohorts (APPPS1 Trem2^−/−^, deltaE9 PLX5622, and PS2APP PLX5622) should be made with caution.

Finally, the validation of the DI approach was performed against SUVR only. We did not include SUV analyses in this study since SUV was shown to have little sensitivity for high-variance datasets [23, 72].

## Conclusions

These translational data provide first evidence that a microglia connectome can be assessed via TSPO-PET imaging in the mammalian brain. Microglia desynchronization is associated with neuropathological hallmarks and cognitive decline in AD and an individual microglia desynchronization index could serve as a personalized immunity biomarker.

## Supplementary Information

Additional file 1: Supplementary Fig. S1. Mean SUV-scaled TSPO-PET uptake in microglia depletion experiment in WT mice. Supplementary Fig. S2. Validation of the depletion experiment by myocardial normalization. Supplementary Fig. S3. Ratio of the number of connections in the investigated mouse cohorts relative to their corresponding reference cohorts. Supplementary Fig. S4. Individual DIs calculated using motor and sensory cortex for cognitively normal and AD continuum subjects. Supplementary Fig. S5. DI compared to SUVR for the study cohorts. Supplementary Fig. S6. Validation analysis using the cognitive dementia ratingindex. Supplementary Fig. S7. Gating strategies and estimation of cellular TSPO tracer uptake beyond microglia and astrocytes. Supplementary Fig. S8. Brain region- and Aβ pathology-specific TSPO expression levels in microglia. Supplementary Fig. S9. Microglia desynchronization in acute ischemic stroke model 7 days after surgery. Supplementary Table S1. Overview on preclinical cohorts. Supplementary Table S2. Overview on training and test cohorts of the human study. Supplementary Table S3. Definition of AD-signature and motor-sensory network regions. Supplementary Table S4. DI compared to SUVR for study cohorts. Supplementary Table S5. Linear fit parameters for DI versus MMSE score for all investigated VOIs. Supplementary Table S6. Linear fit parameters for DI versus CDR SOB for all investigated VOIs

## Data Availability

The data that support the findings of this study are not openly available due to reasons of sensitivity and are available from the corresponding author upon reasonable request. Data are located in controlled access data storage at LMU Munich University Hospital.

## Abbreviations

AD: Alzheimer's disease
ANOVA: Aanalysis of variance
Aβ: Beta-amyloid
BR: Best reference region
BSA: Bovine serum albumin
CDR: Clinical dementia rating
CERAD-NB: Consortium to Establish a Registry for Alzheimer’s Disease Neuropsychological Battery
CSF: Cerebrospinal fluid
CSF1R: Colony-stimulating factor 1 receptor
CT: Computed tomography
CTRL: Healthy control
DAM: Disease-associated microglia
DI: Desynchronization index
FDR: False discovery rate
GLM: Global mean
HAB: High-affinity binder
ICC: Interregional correlation coefficient
LAB: Low-affinity binder
MAB: Mixed-affinity binder
MCI: Mild cognitive impairment
MMSE: Mini-mental state examination
MNI: Montreal Neurological Institute
MX04: Methoxy-X04
PC: Principal component
PCA: Principal component analysis
PET: Positron emission tomography
RMSE: Root mean square error
SCD: Subjective cognitive decline
scRadiotracing: Single-cell radiotracing
SNP: Single nucleotide polymorphisms
SOB: Sum of boxes
SUV: Standardized uptake value
SUVR: Standardized uptake value ratio
TREM2: Triggering receptor expressed on myeloid cells 2
TSPO: 18KDa translocator protein
VOI: Volume of interest
WT: Wild type
μPET: Small-animal positron emission tomography
