# Supplementary material for: Regional desynchronization of microglial activity is associated with cognitive decline in Alzheimer’s disease
Source: Mol Neurodegener. 2024 Sep 5;19:64. doi: 10.1186/s13024-024-00752-6 (PMC11375924; doi:10.1186/s13024-024-00752-6)
Supplement: Supplementary file 1 — Additional file 1: Supplementary Fig. S1. Mean SUV-scaled TSPO-PET uptake in microglia depletion experiment in WT mice. Supplementary Fig. S2. Validation of the depletion experiment by myocardial normalization. Supplementary Fig. S3. Ratio of the number of connections in the investigated mouse cohorts relative to their corresponding reference cohorts. Supplementary Fig. S4. Individual DIs calculated using motor and sensory cortex for cognitively normal and AD continuum subjects. Supplementary Fig. S5. DI compared to SUVR for the study cohorts. Supplementary Fig. S6. Validation analysis using the cognitive dementia ratingindex. Supplementary Fig. S7. Gating strategies and estimation of cellular TSPO tracer uptake beyond microglia and astrocytes. Supplementary Fig. S8. Brain region- and Aβ pathology-specific TSPO expression levels in microglia. Supplementary Fig. S9. Microglia desynchronization in acute ischemic stroke model 7 days after surgery. Supplementary Table S1. Overview on preclinical cohorts. Supplementary Table S2. Overview on training and test cohorts of the human study. Supplementary Table S3. Definition of AD-signature and motor-sensory network regions. Supplementary Table S4. DI compared to SUVR for study cohorts. Supplementary Table S5. Linear fit parameters for DI versus MMSE score for all investigated VOIs. Supplementary Table S6. Linear fit parameters for DI versus CDR SOB for all investigated VOIs [file 13024_2024_752_MOESM1_ESM.pdf]

Supplementary Materials for

Regional Desynchronization of Microglial Activity is Associated with Cognitive Decline in Alzheimer's Disease

Artem Zatcepin et al.

*Corresponding author: Artem Zatcepin. Email: Artem.Zatcepin@med.uni-muenchen.de

This PDF file includes:

Supplementary Figs. S1 to S9
Supplementary Tables S1 to S6

Supplementary Fig. S1

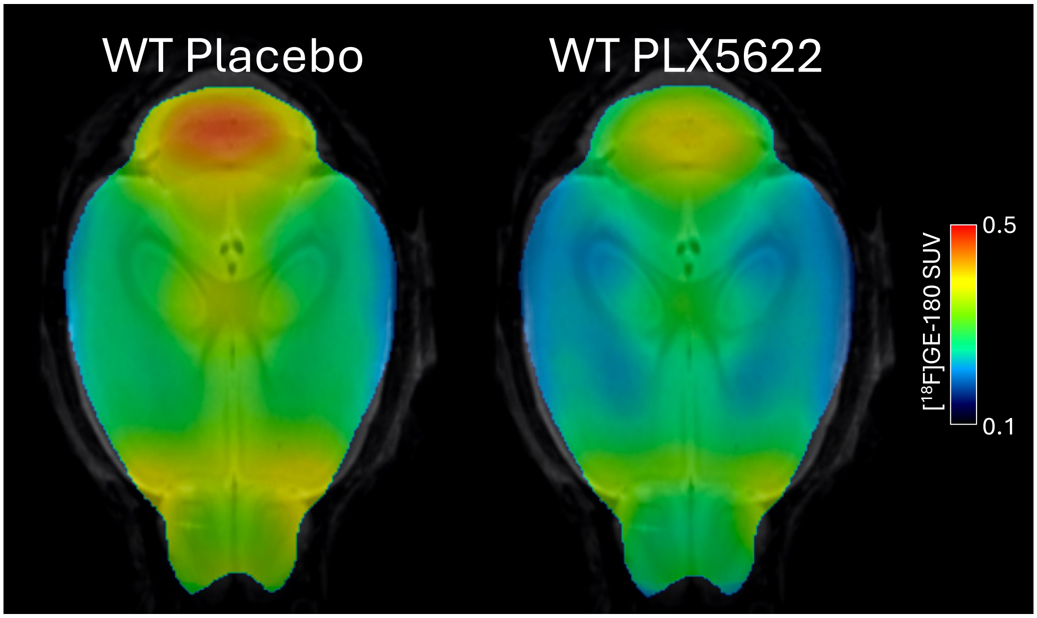

Supplementary Fig. S1. Mean SUV-scaled TSPO-PET uptake in microglia depletion experiment in WT mice. *Left:* mean uptake in mice that received a placebo injection, *right:* mean uptake in mice with microglia depleted using PLX5622.

Supplementary Fig. S2

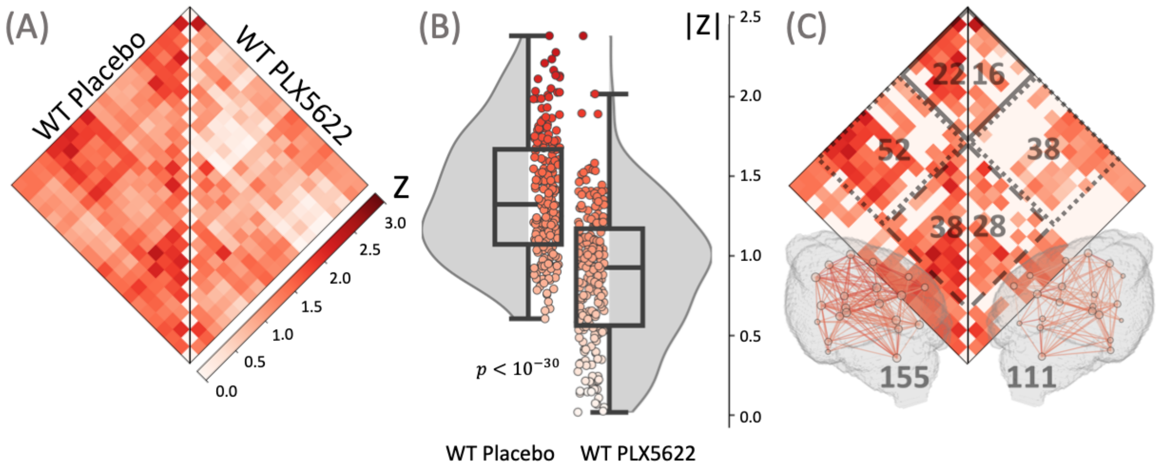

Supplementary Fig. S2. Validation of the depletion experiment by myocardial normalization. Plots show ICC heatmaps **(A)**, absolute ICC values **(B)** and significant connections **(C)** of the depletion experiment with myocardium adjusted standardized uptake values as an alternative normalization method. **(A)** ICC values are shown for all the pairs of the 21 VOIs. **(B)** Distribution of absolute ICCs is shown. **(C)** Significant connections ($p < 0.005$), including cortical (solid line), subcortical (dashed line), and cortical-subcortical (dotted line) connections; the number of the corresponding connections is shown in gray. All significant connections are also projected into 3D brain images, where the color of the connection represents its value; the nodes are individual VOIs, the size of the node reflects the number of its connections; the total number of connections is shown in gray.

Supplementary Fig. S3

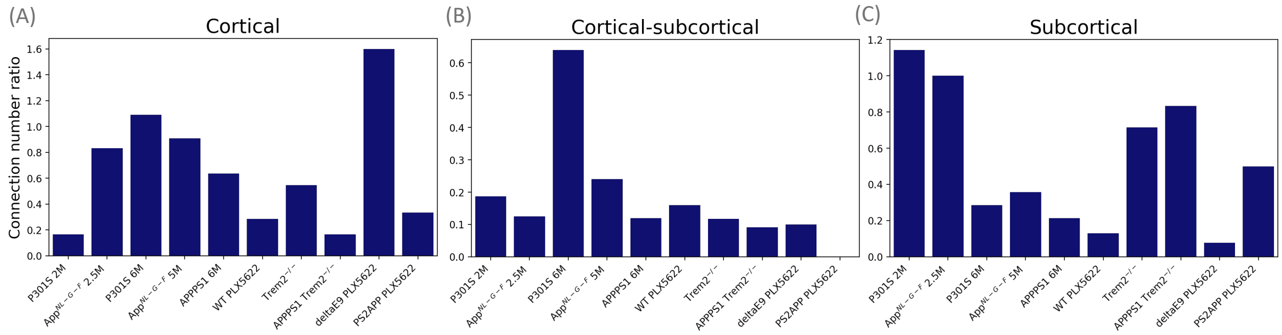

Supplementary Fig. S3. Ratio of the number of connections in the investigated mouse cohorts relative to their corresponding reference cohorts. (**A**) Cortical connections, (**B**) cortical-subcortical connections, (**C**) subcortical connections. The name of each reference cohort can be found in **Supplementary Table S4**.

Supplementary Fig. S4

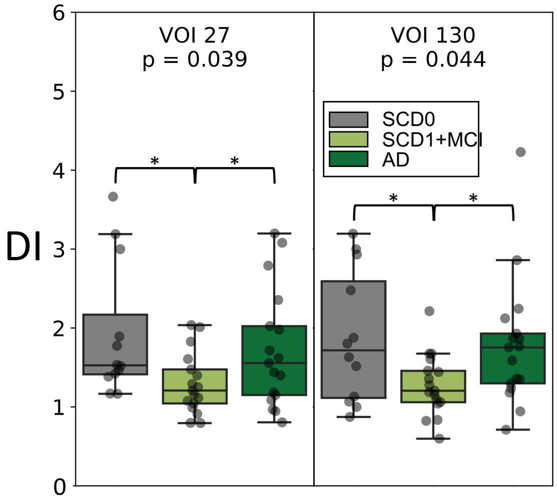

Supplementary Fig. S4. Individual DIs calculated using motor and sensory cortex for cognitively normal and AD continuum subjects. Only VOIs with significant differences in DI are shown. VOI 27 is located in the right postcentral gyrus; VOI 130 is located in the left postcentral gyrus. Unpaired t-test: * $p < 0.05$, ** $p < 0.01$, *** $p < 0.0001$. The p-value of one-way ANOVA is shown on top of each plot.

Supplementary Fig. S5

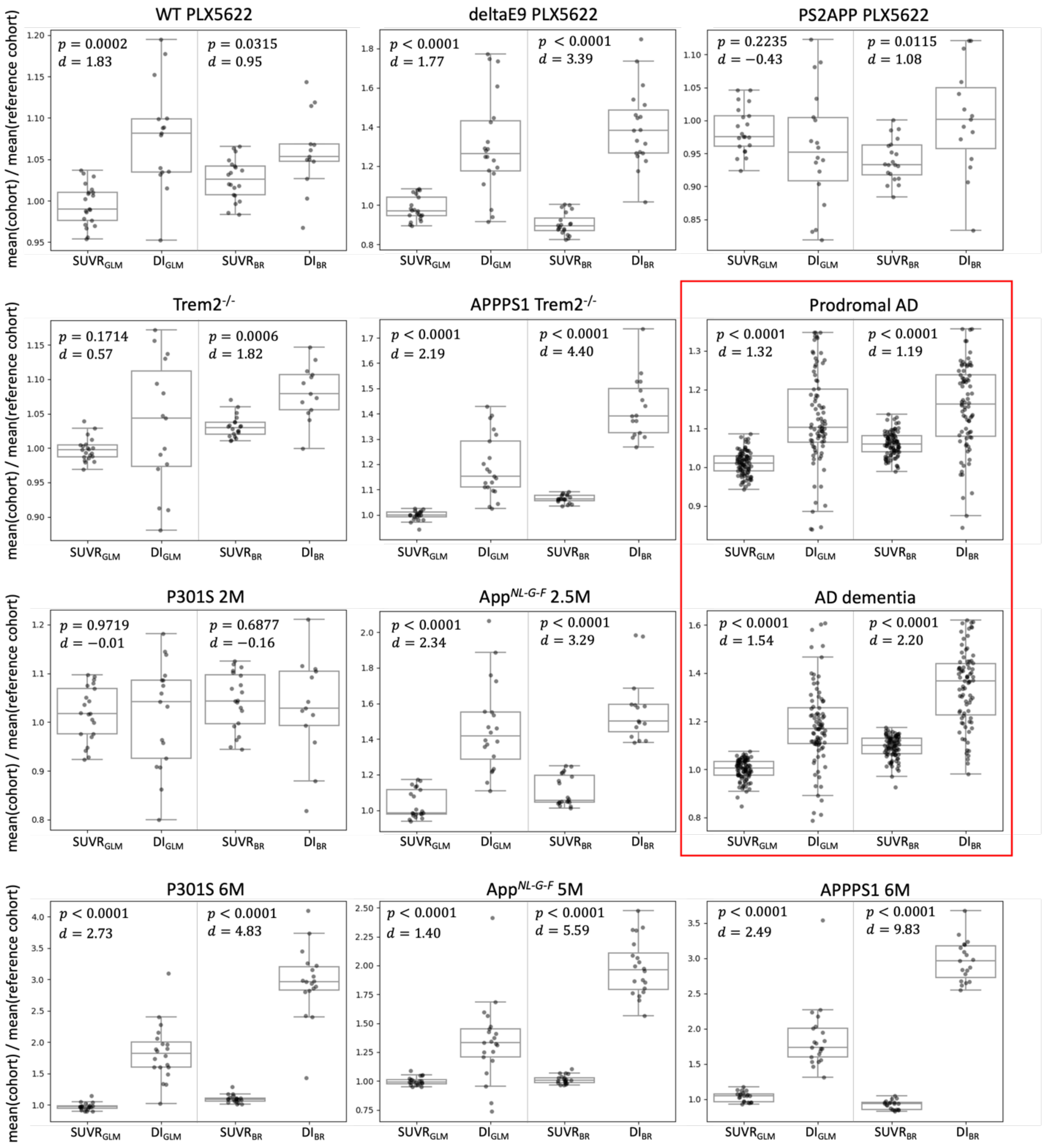

Supplementary Fig. S5. DI compared to SUVR for the study cohorts. The boxplots show the ratio between the mean value (mean SUVR or mean DI) in a cohort relative to its reference cohort (see **Supplementary Table S4** for reference). Every point is a single atlas region. GLM – global mean scaling, BR – best region scaling (see best region list in Supplementary Table S4). Red box indicates the human

cohorts, the rest are the mice cohorts. p is the p-value of the paired two-sided t-test, d is the corresponding value of Cohen’s d (effect size).

Supplementary Fig. S6

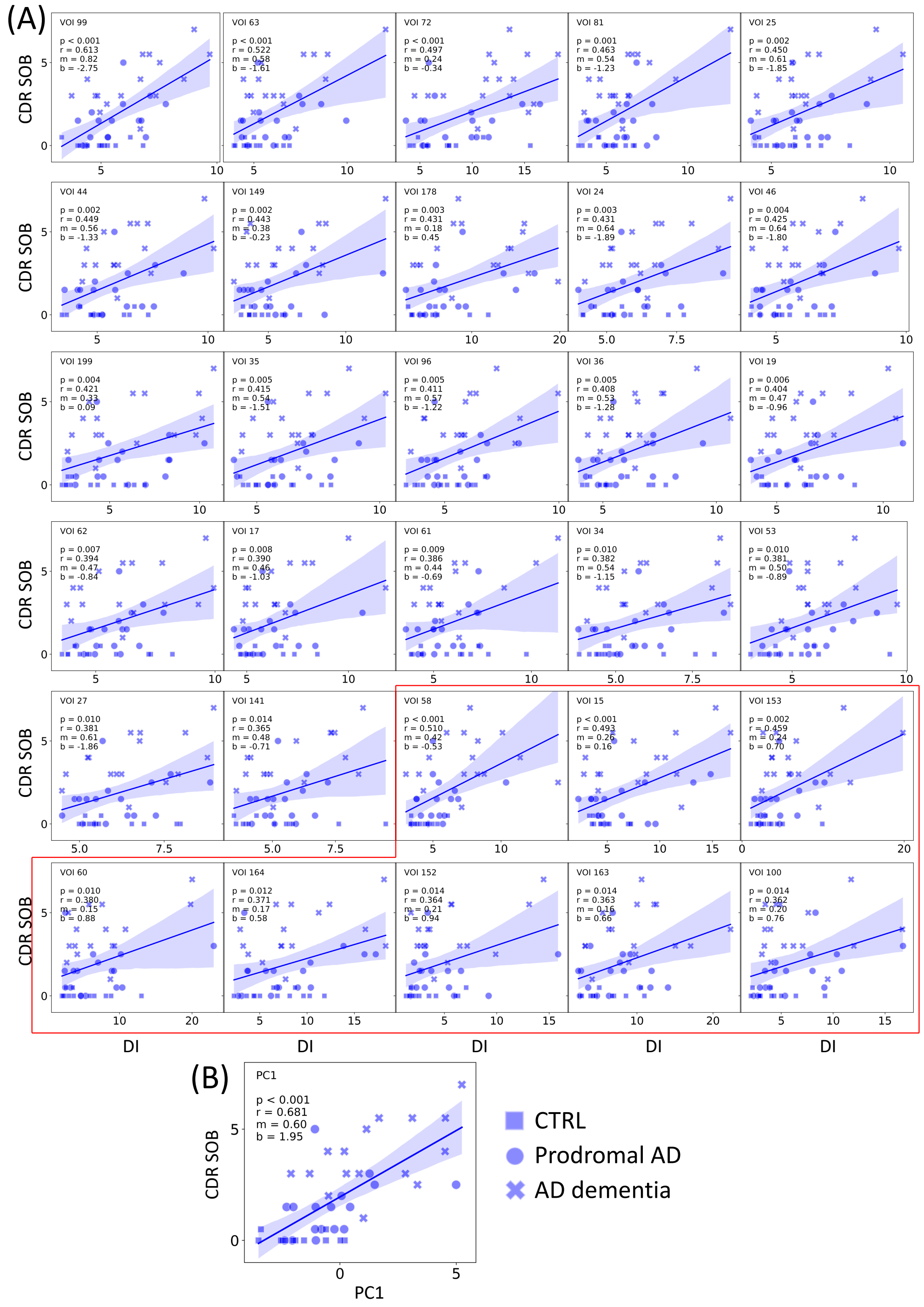

Supplementary Fig. S6. Validation analysis using the cognitive dementia rating (CDR) index. (A) Relationship between DI and CDR sum of boxes (SOB) score for VOIs with significant correlations after FDR correction. Temporal VOIs are indicated by the red box, the rest of the VOIs belong to the parietal lobe. **(B)** Relationship between the first principal component (PC1) calculated based on the VOIs with significant differences in DI compared to CTRL (**Fig. 6**) and CDR SOB. p is the correlation p-value (uncorrected), r is the Pearson's r, m is the slope of the fit, b is its intercept.

Supplementary Fig. S7

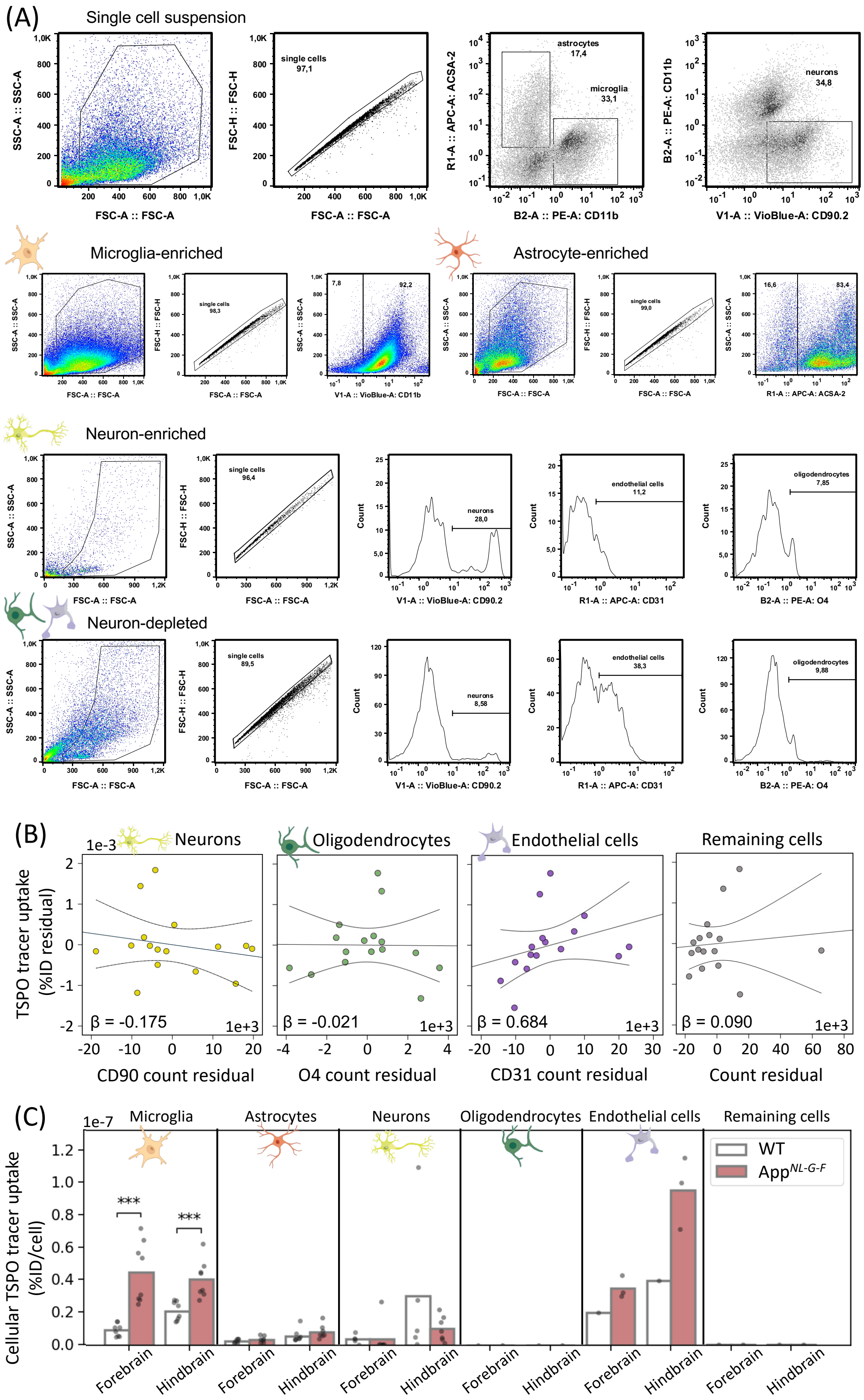

Supplementary Fig. S7. Gating strategies and estimation of cellular TSPO tracer uptake beyond microglia and astrocytes. (A) Upper row shows full-stain analysis of the single cell suspension to determine relative proportions of microglia, astrocytes, and neurons. Panels in the middle show quality control and determination of cell numbers in microglia and astrocyte enriched fractions. Lower two rows show gating strategy of neuron-enriched and neuron-depleted fractions, with a gradient in neuron and endothelial cell fractions. **(B)** Partial correlations between cell count of neurons (CD90.2), endothelial cells (CD31), oligodendrocytes (O4) and remaining cells (negative for all markers) as predictors and radioactivity in cell pellets as dependent variable, derived from regression. β = standardized regression coefficient. **(C)** Estimated cellular TSPO tracer uptake in microglia and astrocytes (both determined per cell in enriched fractions) as well as in neurons, oligodendrocytes, endothelial cells, and remaining cells (all determined by multiple regression). * $p \leq 0.05$, ** $p \leq 0.01$, *** $p \leq 0.001$. Each point in the plots corresponds to a single mouse.

Supplementary Fig. S8

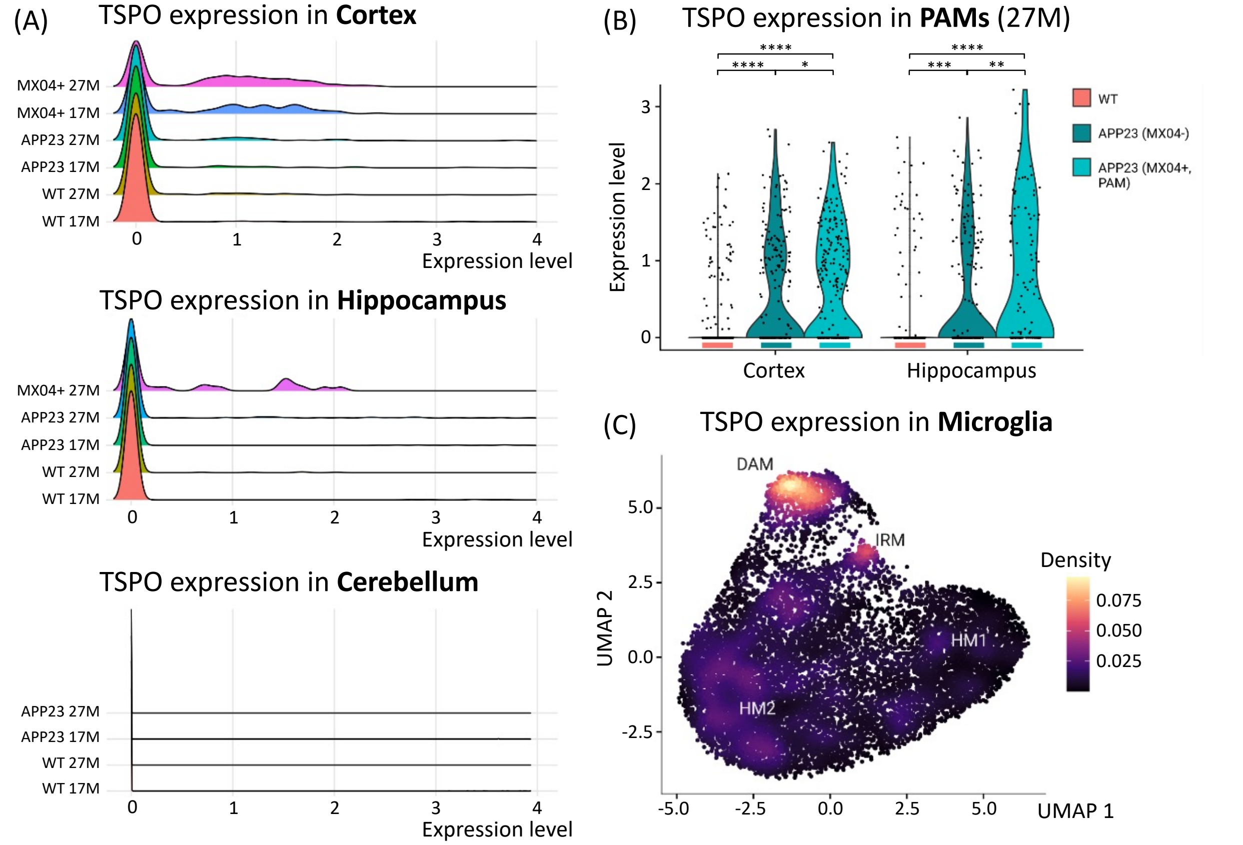

Supplementary Fig. S8. Brain region- and Aβ pathology-specific TSPO expression levels in microglia. **(A)** Ridge plots showing TSPO expression levels according to genotype and age. In the cortex of APP23 animals, where plaque pathology is pronounced, a subpopulation of microglia expresses high levels of TSPO; this population is enriched in plaque-associated microglia (PAMs; MX04+) both in 17- and 27-month-old animals. In the hippocampus, where plaques develop later, a clear increase in TSPO expression only becomes evident in 27-month-old plaque-associated microglial cells. In contrast, no TSPO expression is observed in microglia isolated from the cerebellum, where plaques never develop. **(B)** Violin plot showing TSPO expression in cortical and hippocampal microglia from 27-month-old APP23 and wildtype animals. * $p < 0.05$; ** $p < 0.01$; *** $p < 0.001$; **** $p < 0.0001$. **(C)** Density plot showing strongest enrichment for TSPO expression in microglial subpopulations associated with Aβ-pathology. DAM = Disease associated microglia; IRM = Interferon response microglia; HM = Homeostatic microglia.

Supplementary Fig. S9

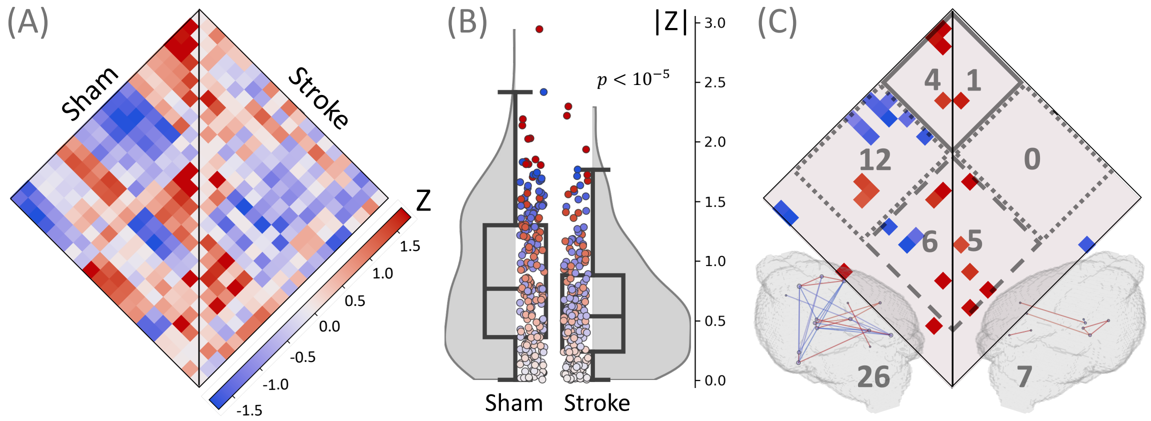

Supplementary Fig. S9. Microglia desynchronization in acute ischemic stroke model 7 days after surgery. Plots show ICC heatmaps, absolute ICC values and significant connections of the sham mice (n=6) and mice with photothrombotic stroke (n=6). **(A)** ICC values for all the pairs of the 21 VOIs. **(B)** Distributions of absolute ICCs, p-values derive from a Wilcoxon signed-rank test. **(C)** Significant connections (p < 0.005), including cortical (solid line), subcortical (dashed line), and cortical-subcortical (dotted line) connections; the number of the corresponding connections is shown in gray. All significant connections are also projected into 3D brain images, where the color of the connection represents its value; the nodes are individual VOIs, the size of the node reflects the number of its connections; the total number of connections is shown in gray.

Supplementary Table S1

Species	Cohort name	Cohort description	Sex	Number of subjects	Age, months
Mouse	WT Placebo	WT with placebo treatment for seven weeks	Female	14	6-9
Mouse	WT PLX5622	WT with PLX5622 treatment for seven weeks	Female	14	6-9
Mouse	TREM2$^{+/+}$	WT with intact TREM2 gene	Female	17	12
Mouse	TREM2$^{-/-}$	WT with TREM2 knock-out	Female	17	12
Mouse	APPPS1 Trem2$^{+/+}$	Transgenic Aβ mouse model with intact TREM gene	Female	11	12
Mouse	APPPS1 Trem2$^{-/-}$	Transgenic Aβ mouse model with TREM2 knock-out	Female	11	12
Mouse	WT 2M	WT	Female	12	2
Mouse	P301S 2M	Tau mouse model at earliest stage of tau aggregation	Female	12	2
Mouse	App$^{NL\text{-}G\text{-}F}$ 2.5M	APP knock-in Aβ mouse model at earliest stage of plaque deposition	Female	12	2.5
Mouse	WT 6M	WT	Female	14	6
Mouse	P301S 6M	Tau mouse model at a stage of established tau aggregation	Female	14	6
Mouse	App$^{NL\text{-}G\text{-}F}$ 5M	APP knock-in Aβ mouse model at a stage of moderate plaque deposition	Female	14	5
Mouse	APPPS1 6M	Transgenic Aβ mouse model at a stage of moderate plaque deposition	Female	14	6
Mouse	PS2APP Placebo	Transgenic Aβ mouse model with placebo treatment for seven weeks	Female	10	11.5
Mouse	PS2APP PLX5622	Transgenic Aβ mouse model with PLX5622 treatment for seven weeks	Female	10	11.5
Mouse	deltaE9 Placebo	Transgenic Aβ mouse model with placebo treatment for four weeks	Female	8	5.5-6.5
Mouse	deltaE9 PLX5622	Transgenic Aβ mouse model with PLX5622 treatment for four weeks	Female	8	5.5-6.5
Mouse	Sham	WT mice 7d after sham surgery	Male	6	2-2.5

Mouse	Stroke	WT mice 7 days after photothrombotic stroke surgery	Male	6	2-2.5
Mouse	WT (scRadiotracing)	WT	Female	8	6-7
Mouse	$App^{NL\text{-}G\text{-}F}$ (scRadiotracing)	APP knock-in Aβ mouse model	Female	8	6-7

Supplementary Table S1. Overview on preclinical cohorts. Shading shows cohorts that were compared to each other.

Supplementary Table S2

	CTRL train	CTRL test	Prodromal AD	AD dementia	ANOVA F-value (p-value)
n	13	12	17	17	
Age (years)	42.7 ± 19.4	72.7 ± 4.2	68.5 ± 7.3	71.3 ± 7.3	22.97 ($<10^{-9}$)
Sex	♀ 11 / ♂ 2	♀ 7 / ♂ 5	♀ 7 / ♂ 10	♀ 13 / ♂ 4	2.71 (0.05)
rs6971 SNP	MAB: 8<br>HAB: 5<br>(n=13)	MAB: 8<br>HAB: 4<br>(n=12)	MAB: 10<br>HAB: 7<br>(n=17)	MAB: 13<br>HAB: 4<br>(n=17)	1.02 (0.39)
rs429358 ApoE1 pos 112	CC: 0<br>CT: 2<br>TT: 1<br>(n=3)	CC: 0<br>CT: 2<br>TT: 10<br>(n=12)	CC: 2<br>CT: 3<br>TT: 3<br>(n=8)	CC: 2<br>CT: 7<br>TT: 3<br>(n=12)	3.45 (0.03)
rs7412 ApoE2 pos 158	CC: 3<br>CT: 0<br>(n=3)	CC: 10<br>CT: 2<br>(n=12)	CC: 8<br>CT: 0<br>(n=8)	CC: 12<br>CT: 0<br>(n=12)	1.36 (0.27)
sTREM2	n.a.	10.9 ± 3.1<br>(n=6)	10.2 ± 2.0<br>(n=7)	11.2 ± 3.5<br>(n=10)	0.49 (0.07)
MMSE	29.5 ± 0.6<br>(n=4)	29.1 ± 1.1<br>(n=12)	27.9 ± 1.7<br>(n=17)	22.7 ± 2.6<br>(n=17)	36.49 ($<10^{-11}$)
CDR SOB	0.13 ± 0.25<br>(n=4)	0.08 ± 0.20<br>(n=12)	1.44 ± 1.31<br>(n=17)	3.81 ± 1.56<br>(n=16)	26.41 ($<10^{-9}$)

Supplementary Table S2. Overview on training and test cohorts of the human study. CTRL = healthy control; AD = Alzheimer's disease; ANOVA = analysis of variance; SNP = single nucleotide polymorphism; ApoE = Apolipoprotein E; sTREM2 = soluble triggering receptor expressed on myeloid cells type 2; MMSE = mini-mental-state-examination; CDR SOB = cognitive dementia rating sum of boxes

Supplementary Table S3

Network	Region	Schaefer VOIs
**AD signature**	Parietal lobe	6, 17, 19, 23, 24, 25, 27, 33 , 34, 35, 36, 37, 38, 39, 40, 44, 45, 46, 53, 61, 62, 63, 72, 79, 81, 82, 96, 97, 98, 99, 107, 110, 115, 119, 121, 124, 127, 128, 130, 132, 137, 138, 139, 140, 141, 142, 143, 144, 149, 150, 157, 165, 166, 167, 177, 178, 182, 183, 184, 198, 199, 200
	Temporal lobe	15, 16, 31, 32, 57, 58, 59, 60, 64, 74, 75, 76, 77, 78, 100, 101, 102, 116, 117, 135, 148, 152, 153, 162, 163, 164, 168, 185, 186, 187, 188, 189
**Motor-sensory**	Precentral gyrus	21, 26, 30, 43, 129, 145, 151
	Supplementary motor area	54, 95, 126, 158
	Paracentral lobule	29, 133
	Postcentral gyrus	20, 23, 24, 25, 27, 122, 124, 125, 127, 128, 130, 134, 137, 139

Supplementary Table S3. Definition of AD-signature and motor-sensory network regions. Volume of interest (VOI) numbers are provided for all included regions.

Supplementary Table S4

**Cohort**	**Reference cohort**	**p-value DI_{GLM} vs $SUVR_{GLM}$**	**Cohen's d DI_{GLM} vs $SUVR_{GLM}$**	**p-value DI_{BR} vs $SUVR_{BR}$**	**Cohen's d DI_{BR} vs $SUVR_{BR}$**	**Best region (BR) name**
WT PLX5622	WT Placebo	0.0002	1.83	0.0315	0.95	Cerebellum
deltaE9 PLX5622	deltaE9 Placebo	<0.0001	1.77	<0.0001	3.39	Brain stem
PS2APP PLX5622	PS2APP Placebo	0.2235	-0.43	0.0115	1.08	Brain stem
$Trem2^{-/-}$	$Trem2^{+/+}$	0.1714	0.57	0.0006	1.82	Thalamus left
APPPS1 $Trem2^{-/-}$	APPPS1 $Trem2^{+/+}$	<0.0001	2.19	<0.0001	4.40	Auditory cortex right
P301S 2M	WT 2M	0.9719	-0.01	0.6877	-0.16	Brain stem
App^{NL-G-F} 2.5M	WT 2M	<0.0001	2.34	<0.0001	3.29	Brain stem
P301S 6M	WT 6M	<0.0001	2.73	<0.0001	4.83	Somatosensory cortex right
App^{NL-G-F} 5M	WT 6M	0.0001	1.40	<0.0001	5.59	Somatosensory cortex right
APPPS1 6M	WT 6M	<0.0001	2.49	<0.0001	9.83	Somatosensory cortex right
Prodromal AD	CTRL test	<0.0001	1.32	<0.0001	1.19	Schaefer 24
AD dementia	CTRL test	<0.0001	1.54	<0.0001	2.20	Schaefer 24

Supplementary Table S4. DI compared to SUVR for study cohorts. GLM – global mean scaling, BR – best region scaling.

Supplementary Table S5

Region	Intercept	Slope	r	p	p_{FDR}
**58**	**30.9356525**	**-0.7903221**	**-0.5446341**	**9.1118E-05**	**0.00856505**
**72**	**30.4827304**	**-0.4355204**	**-0.5210918**	**0.00020478**	**0.00962485**
**153**	**28.7173935**	**-0.460459**	**-0.5064532**	**0.00032911**	**0.01031198**
**178**	**29.1236188**	**-0.3401915**	**-0.4641679**	**0.00115667**	**0.02718176**
**99**	**32.5858166**	**-1.0955367**	**-0.4635156**	**0.00117786**	**0.02214382**
149	29.9731419	-0.6514939	-0.4251488	0.00322653	0.05054892
**199**	**29.4655864**	**-0.5632666**	**-0.4230137**	**0.0034013**	**0.04567455**
152	28.2217828	-0.3971198	-0.4058564	0.00513484	0.06033436
61	30.8376741	-0.7506634	-0.3946425	0.00664634	0.0694173
121	29.0570229	-0.395672	-0.391596	0.00711838	0.06691277
96	31.4689874	-0.9229614	-0.382642	0.0086777	0.07415485
15	28.7163243	-0.358487	-0.3808446	0.00902394	0.07068755
34	31.4769068	-0.9082752	-0.362133	0.013395	0.09685616
24	31.9522884	-0.9390339	-0.3620483	0.01341831	0.09009434
63	30.6117109	-0.7080303	-0.3595632	0.01411747	0.0884695
27	32.631063	-1.0114729	-0.3588963	0.0143103	0.08407302
150	29.254826	-0.5760147	-0.3581167	0.01453854	0.08038957
44	30.891446	-0.7887625	-0.35609	0.01514644	0.07909808
81	30.593627	-0.7316611	-0.3556492	0.0152815	0.0756032
25	31.5995283	-0.8552708	-0.3553469	0.0153747	0.07226107
64	28.6446634	-0.4553819	-0.3553323	0.01537922	0.06884031
163	28.5103653	-0.2828606	-0.3552258	0.01541216	0.06585198
62	30.6411167	-0.7390494	-0.3494415	0.0172946	0.0706823
157	29.2317563	-0.5269459	-0.3405865	0.02055164	0.0804939
110	29.1877971	-0.4286028	-0.3374036	0.02184202	0.08212598
97	30.30276	-0.7007449	-0.3308979	0.02469195	0.08927089
60	27.85652	-0.2289824	-0.3180232	0.03125256	0.10880522
36	30.687463	-0.7238685	-0.3139604	0.03360042	0.11280141
46	31.1628132	-0.8342803	-0.3126286	0.03440102	0.11150675
23	31.0910477	-0.767319	-0.3122518	0.03463038	0.10850852
17	30.460663	-0.6503287	-0.3097701	0.03617263	0.10968474
35	30.6742558	-0.6918098	-0.2978606	0.04438009	0.13036651
128	29.4756865	-0.4793251	-0.2972416	0.04484504	0.12774042
19	30.0290492	-0.6004747	-0.2940231	0.04732694	0.13084507
53	30.0910173	-0.6616066	-0.2923973	0.04862236	0.13058576
162	28.1006796	-0.2618433	-0.2916502	0.04922721	0.12853773
141	30.0236593	-0.6735091	-0.2900856	0.05051367	0.12833201
98	29.6385615	-0.5174567	-0.2871168	0.0530294	0.131178
74	28.0672182	-0.279582	-0.2789492	0.06047518	0.14576069
33	30.0643601	-0.5869175	-0.2694296	0.07018444	0.16493344
119	29.2832372	-0.4886748	-0.2671438	0.07269039	0.16665602
79	29.4017189	-0.5164392	-0.262044	0.07853617	0.17577143
177	28.2393617	-0.3501029	-0.2552796	0.08685247	0.18986355
102	27.5943651	-0.2345449	-0.2547086	0.08758472	0.1871128
45	29.4008726	-0.518773	-0.233867	0.11774906	0.2459647
130	28.7060486	-0.3732233	-0.2310986	0.12228509	0.24988692
189	27.4866959	-0.2132722	-0.2223825	0.13742866	0.27485731
198	28.1367951	-0.3418839	-0.2186673	0.14429175	0.28257134
39	29.1997679	-0.4270309	-0.2177821	0.14596383	0.28001224
167	27.7657052	-0.2907528	-0.2034404	0.17508082	0.32915194
168	27.743793	-0.3100178	-0.202413	0.17731674	0.32681909
127	28.8960239	-0.4317138	-0.2014026	0.17953546	0.32454487
164	27.498498	-0.1501674	-0.1894462	0.20731539	0.36769145
6	28.6291292	-0.3337553	-0.1835304	0.22212022	0.38665371
78	24.992992	0.30538126	0.17824004	0.23596513	0.40328586
187	25.0740578	0.26692945	0.17238472	0.25196277	0.42293751
139	28.5365048	-0.4004626	-0.1662161	0.2695891	0.44458553
185	27.0626535	-0.1173273	-0.1631517	0.27864207	0.45159232
57	27.3501292	-0.1265963	-0.1631276	0.27871403	0.44405286
32	27.2273147	-0.1816098	-0.1526298	0.31122949	0.48759288
101	27.1866498	-0.1394166	-0.1502595	0.31889238	0.49140793
117	27.2957386	-0.1849629	-0.1483604	0.32511714	0.49291953
188	26.9757239	-0.137941	-0.1423476	0.34532508	0.51524694

76	25.4040859	0.18258159	0.14092522	0.35021591	0.51437962
40	27.9692832	-0.2363412	-0.1383467	0.35919	0.519444
100	27.0736027	-0.1318155	-0.1378348	0.3609879	0.51413428
77	27.2623178	-0.1537046	-0.1369523	0.36410047	0.51082752
135	25.5112344	0.11924736	0.13641557	0.36600124	0.50594289
37	28.15043	-0.2691805	-0.1341539	0.37407682	0.50961189
138	27.440721	-0.1819768	-0.1312746	0.38451	0.516342
132	27.4844684	-0.1716359	-0.1306072	0.38695263	0.51230348
183	27.2852323	-0.2014453	-0.128898	0.39324945	0.51340901
82	27.648506	-0.2338104	-0.1245841	0.40940692	0.52718151
124	27.7492934	-0.2306646	-0.1186126	0.43239055	0.54925286
107	27.4739943	-0.1812577	-0.1176935	0.43599061	0.54644157
144	27.1534455	-0.1047963	-0.1157157	0.44379457	0.54890382
200	27.2069393	-0.1670852	-0.1084754	0.47300979	0.57744052
143	25.416806	0.12654911	0.1048674	0.4879409	0.58803134
75	27.0558088	-0.1215342	-0.1015561	0.50185715	0.59714648
115	27.3505031	-0.1852538	-0.0883843	0.55915339	0.65700523
148	26.7889938	-0.0956412	-0.0869391	0.56562175	0.65640055
137	27.0560502	-0.140429	-0.0812231	0.59153771	0.6781042
140	25.6056308	0.08841912	0.07656327	0.61304623	0.69429332
165	26.9773467	-0.1088816	-0.0737996	0.62595805	0.70047686
38	27.0597216	-0.113058	-0.0651289	0.66717641	0.73781862
186	26.8409446	-0.1058772	-0.0602129	0.69099462	0.75527319
182	26.6973309	-0.0699509	-0.0492568	0.74512079	0.80507303
31	26.6982468	-0.0616684	-0.0459126	0.76190186	0.81384972
59	26.0647586	0.02672254	0.03526913	0.81599739	0.86183994
184	26.529276	-0.0361046	-0.0310468	0.83770974	0.87494129
16	26.4967809	-0.0340768	-0.0211239	0.88918004	0.91849367
116	26.2553975	0.00972065	0.00849595	0.95531194	0.97607959
142	26.2249227	0.01263649	0.00810395	0.95737189	0.96766621
166	26.3484177	-0.0071623	-0.0073629	0.96126666	0.96126666

Supplementary Table S5. Linear fit parameters for DI versus MMSE score for all investigated VOIs. r is the Pearson's r, p is the uncorrected p-value, p_{FDR} is the p-value after FDR correction. Significant correlations ($p < 0.05$) are shown in italics, correlations that remained significant after FDR correction ($p_{FDR} < 0.05$) are shown in bold. The regions are sorted according to their p-value.

Supplementary Table S6

Region	Intercept	Slope	r	p	p_{FDR}
**99**	**-2.75186**	**0.817361**	**0.612738**	**7.68E-06**	**0.000722**
**63**	**-1.60849**	**0.583716**	**0.521634**	**0.000238**	**0.011195**
**58**	**-0.53454**	**0.41749**	**0.509781**	**0.000348**	**0.010892**
**72**	**-0.34377**	**0.239384**	**0.497127**	**0.000513**	**0.01205**
**15**	**0.158665**	**0.261391**	**0.492987**	**0.00058**	**0.010912**
**81**	**-1.22868**	**0.540065**	**0.463241**	**0.001353**	**0.021193**
**153**	**0.698132**	**0.236384**	**0.458927**	**0.00152**	**0.020411**
**25**	**-1.85355**	**0.61108**	**0.450211**	**0.001915**	**0.022498**
**44**	**-1.33294**	**0.561131**	**0.449081**	**0.001972**	**0.020597**
**149**	**-0.22755**	**0.382198**	**0.44296**	**0.00231**	**0.021711**
**178**	**0.447901**	**0.180252**	**0.43143**	**0.003087**	**0.026377**
**24**	**-1.88896**	**0.636629**	**0.431409**	**0.003088**	**0.024191**
**46**	**-1.79519**	**0.638079**	**0.424707**	**0.003639**	**0.02631**
**199**	**0.087913**	**0.334197**	**0.421289**	**0.003951**	**0.026529**
**35**	**-1.50633**	**0.544188**	**0.41502**	**0.004586**	**0.028737**
**96**	**-1.22076**	**0.565106**	**0.410612**	**0.005084**	**0.029866**
**36**	**-1.28381**	**0.530343**	**0.408184**	**0.005378**	**0.029734**
**19**	**-0.96077**	**0.465134**	**0.404499**	**0.005852**	**0.030559**
**62**	**-0.84496**	**0.474401**	**0.394108**	**0.00739**	**0.036561**
**17**	**-1.02856**	**0.463191**	**0.390478**	**0.008004**	**0.037621**
**61**	**-0.68674**	**0.438361**	**0.386336**	**0.008759**	**0.039205**
**34**	**-1.14886**	**0.540344**	**0.382083**	**0.009596**	**0.041001**
**53**	**-0.89422**	**0.496048**	**0.381115**	**0.009796**	**0.040035**
**27**	**-1.86349**	**0.606727**	**0.380995**	**0.009821**	**0.038465**
**60**	**0.875561**	**0.154045**	**0.379947**	**0.010042**	**0.037758**
**164**	**0.577487**	**0.16729**	**0.371421**	**0.012004**	**0.043398**
**141**	**-0.71498**	**0.477666**	**0.365454**	**0.013564**	**0.047223**
**152**	**0.939831**	**0.210189**	**0.363943**	**0.013986**	**0.046951**
**163**	**0.655928**	**0.163255**	**0.363371**	**0.014148**	**0.045859**
**100**	**0.762834**	**0.196541**	**0.362433**	**0.014418**	**0.045175**
128	-0.2148	0.322358	0.354786	0.016785	0.050898
121	0.520057	0.200636	0.352019	0.01772	0.052052
119	-0.27401	0.359581	0.348857	0.018841	0.053667
23	-1.07852	0.482196	0.347612	0.019298	0.053354
98	-0.37102	0.359561	0.344479	0.020491	0.055033
97	-0.4064	0.409094	0.342574	0.021246	0.055476
157	0.269153	0.29798	0.342022	0.021469	0.054544
150	0.352913	0.307553	0.338987	0.022732	0.056231
127	-0.54111	0.409477	0.338848	0.022791	0.054932
45	-0.57095	0.417832	0.334586	0.024672	0.05798
102	0.980778	0.171897	0.331301	0.026209	0.06009
79	-0.14799	0.3471	0.310187	0.038108	0.085288
82	0.052491	0.324576	0.30693	0.040286	0.088068
189	1.014369	0.163286	0.302304	0.043555	0.09305
130	0.17024	0.271503	0.298138	0.046681	0.097511
33	-0.41963	0.36732	0.296847	0.047686	0.097445
64	0.832397	0.215139	0.292918	0.050852	0.101703
177	0.68953	0.224466	0.289388	0.05384	0.105436
110	0.550679	0.204127	0.285383	0.057399	0.110112
167	0.788331	0.226619	0.28109	0.061421	0.115471
78	3.067248	-0.26685	-0.27665	0.065813	0.121303
101	1.012948	0.143758	0.275222	0.067278	0.121618
148	1.078374	0.165111	0.265403	0.078065	0.138454
6	0.032885	0.272435	0.264494	0.079128	0.137742
168	0.898293	0.219136	0.253129	0.093405	0.159637
183	0.832597	0.222449	0.251811	0.095182	0.159769
138	0.730107	0.189905	0.242352	0.108711	0.179277
39	0.110908	0.267274	0.242111	0.109074	0.176774
137	0.760683	0.216986	0.22294	0.14101	0.224661
57	1.102008	0.098214	0.222604	0.141626	0.221881
37	0.221808	0.247838	0.219397	0.147601	0.227451
166	1.212657	0.115969	0.211303	0.163509	0.247901
188	1.368116	0.115497	0.20981	0.166574	0.248539
135	2.592735	-0.10284	-0.20351	0.179979	0.264345

139	0.426192	0.268411	0.197899	0.192533	0.278432
74	1.224526	0.109895	0.194044	0.201518	0.28701
182	1.099849	0.145828	0.182077	0.231277	0.324479
40	0.68178	0.177389	0.181572	0.232596	0.32153
115	0.800081	0.198663	0.168365	0.268918	0.366352
186	1.11761	0.158033	0.159118	0.296472	0.398119
165	1.116726	0.129512	0.154939	0.309503	0.409765
124	0.854896	0.171789	0.153808	0.31309	0.408756
38	0.971657	0.143042	0.145245	0.341109	0.439236
198	1.256553	0.124437	0.141306	0.354502	0.450314
132	1.200375	0.106656	0.13834	0.364796	0.457211
142	1.166573	0.119685	0.135822	0.373673	0.462175
75	1.368112	0.090863	0.132099	0.387034	0.472483
32	1.474719	0.089241	0.131514	0.389158	0.468985
107	1.21624	0.110004	0.126143	0.408981	0.486636
184	1.402998	0.084757	0.12568	0.410717	0.482592
185	1.613441	0.048469	0.118675	0.437486	0.507699
162	1.533433	0.056729	0.112235	0.46293	0.530676
187	2.325512	-0.08696	-0.09936	0.516093	0.584491
200	1.464725	0.084571	0.097506	0.523991	0.586371
31	1.45292	0.07321	0.096638	0.527709	0.583584
16	1.434488	0.086359	0.095095	0.534354	0.584062
144	1.630874	0.036285	0.070556	0.645115	0.69702
143	2.150469	-0.03236	-0.04742	0.757089	0.808709
140	2.057212	-0.01696	-0.02584	0.866204	0.914867
117	1.84915	0.013779	0.019437	0.899154	0.939117
77	1.845649	0.01243	0.019417	0.899259	0.928905
76	1.894375	0.0056	0.007634	0.960303	0.98118
116	1.912649	0.001895	0.002941	0.984702	0.99529
59	1.913286	0.000995	0.002333	0.987865	0.987865

Supplementary Table S6. Linear fit parameters for DI versus CDR SOB for all investigated VOIs. r is the Pearson's r, p is the uncorrected p-value, p_{FDR} is the p-value after FDR correction. Significant correlations ($p < 0.05$) are shown in italics, correlations that remained significant after FDR correction ($p_{FDR} < 0.05$) are shown in bold. The regions are sorted according to their p-value.
